# INF2-mediated actin filament reorganization confers intrinsic resilience to neuronal ischemic injury

**DOI:** 10.1038/s41467-022-33268-y

**Published:** 2022-10-13

**Authors:** Barbara Calabrese, Steven L. Jones, Yoko Shiraishi-Yamaguchi, Michael Lingelbach, Uri Manor, Tatyana M. Svitkina, Henry N. Higgs, Andy Y. Shih, Shelley Halpain

**Affiliations:** 1 Department of Neurobiology, School of Biological Sciences, University of California, San Diego, and Sanford Consortium for Regenerative Medicine, La Jolla, CA 92093 USA; 2 Department of Biology, University of Pennsylvania, Philadelphia, PA 19104-4544 USA; 3 Japan Science and Technology Agency (JST), Tokyo, 102-8666 Japan; 4 Neurosciences Interdepartmental Program, Stanford University, Stanford, CA 94305 USA; 5 The Salk Institute for Biological Studies, La Jolla, CA 92037 USA; 6 Department of Biochemistry, Geisel School of Medicine, Hanover, NH 03755 USA; 7 Center for Developmental Biology and Regenerative Medicine, Seattle Children’s Research Institute, Seattle, WA 98101 USA; 8 Department of Neuroscience, Medical University of South Carolina, Charleston, SC USA

**Keywords:** Cell death in the nervous system, Actin

## Abstract

During early ischemic brain injury, glutamate receptor hyperactivation mediates neuronal death via osmotic cell swelling. Here we show that ischemia and excess NMDA receptor activation cause actin to rapidly and extensively reorganize within the somatodendritic compartment. Normally, F-actin is concentrated within dendritic spines. However, <5 min after bath-applied NMDA, F-actin depolymerizes within spines and polymerizes into stable filaments within the dendrite shaft and soma. A similar actinification occurs after experimental ischemia in culture, and photothrombotic stroke in mouse. Following transient NMDA incubation, actinification spontaneously reverses. Na^+^, Cl^−^, water, and Ca^2+^ influx, and spine F-actin depolymerization are all necessary, but not individually sufficient, for actinification, but combined they induce activation of the F-actin polymerization factor inverted formin-2 (INF2). Silencing of INF2 renders neurons vulnerable to cell death and INF2 overexpression is protective. Ischemia-induced dendritic actin reorganization is therefore an intrinsic pro-survival response that protects neurons from death induced by cell edema.

## Abstract (web summary)

Post injury cytoskeletal modifications in neurons are not fully understood. Here the authors describe a pro-survival actin cytoskeletal reorganization in neurons triggered during a model of ischemic stroke.

## Introduction

Ischemic stroke results from occlusion of cerebral blood vessels, causing brain tissue injury due to loss of oxygen and glucose supply^1^. It is a leading cause of death and chronic disability, and has enormous public health implications, especially recently in the context of coronavirus disease-2019, which increases a severely ill patient’s risk of stroke^2–5^. Beyond anticoagulant therapies, there are relatively few emergency treatment options for stroke. This highlights the need for a deeper understanding of the biological cascades ensuing from an ischemic event, including cellular pro-survival mechanisms that could minimize the ultimate brain tissue damage.

Although apoptosis accounts for much of the delayed neuronal death that occurs over days and weeks following a stroke, most of the neuronal cell death that occurs in the early hours is due to a pathological swelling of neurons that leads to disruption of plasma membrane integrity^6–9^. Neuronal swelling, which is also called cytotoxic edema, is triggered when catastrophic ATP depletion perturbs ionic balance, leading to a massive influx of ions through multiple entry routes, with cation entry through *N*-methyl-d-aspartate receptors (NMDARs) and voltage-dependent channels and chloride entry through the SLC26A11 ion exchanger playing major roles^10^. Neuronal depolarization spreads in waves from the site of initial ischemia via local release of glutamate, thereby exacerbating and spreading the initial damage^11^. NMDA receptor hyperactivation plays an especially critical role, as administration of NMDA receptor antagonists before or even just after the onset of ischemia results in significantly reduced infarct volume in experimental models^11–13^.

Neurons, like most other cells, respond to cellular stress and injury, including ischemic injury, by mounting a series of pro-survival responses, with changes in organelles, reduction in protein synthesis, and activation of pro-survival genes^14^. In contrast, the pro-survival functions of the cytoskeleton are less well characterized. In the present work we describe an extensive reorganization of neuronal filamentous actin (F-actin) that occurs in response to stroke, oxygen and glucose deprivation, or NMDA receptor hyperactivation. F-actin is rapidly depolymerized within dendritic spines^14–16^, and, as we demonstrate here, it simultaneously polymerizes extensively within the soma and dendrites. This result is surprising given the usual ATP dependence of actin polymerization and the reduced ATP availability during ischemia. We find that this F-actin response is pro-survival and selectively triggered by conditions that elicit neuronal cytotoxic edema. The F-actin build-up in the soma and dendrites results in long, slowly turning over actin filaments that persist while the stress is present. However, the F-actin reorganization spontaneously reverses if the stress is transient. We demonstrate that activation of inverted formin-2 (INF2) is a key mediator of this neuronal pro-survival cytoskeletal response.

## Results

### Actin reorganization is induced by oxygen and glucose deprivation in vitro

**Fig. 1:**
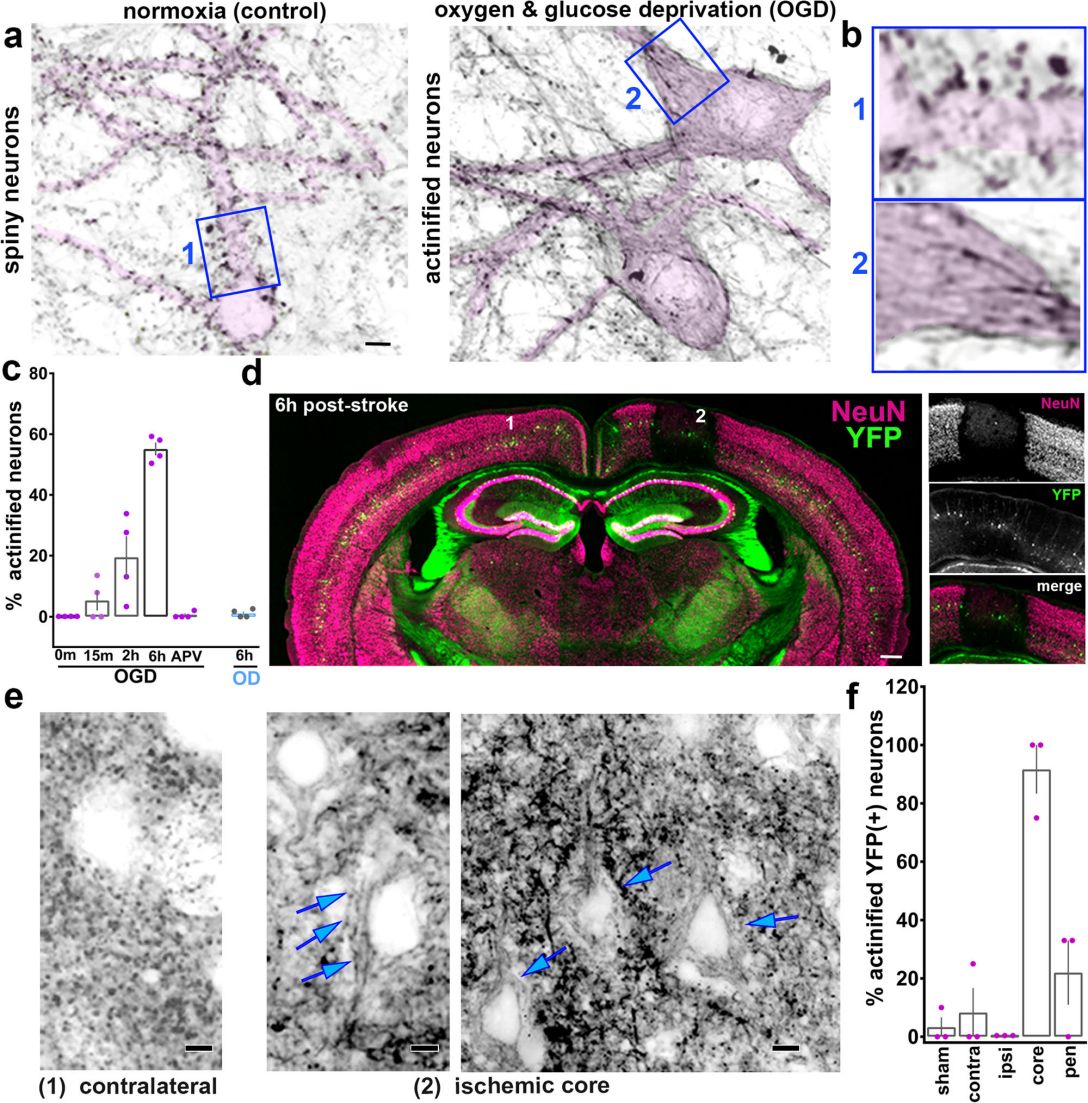
Ischemia-induced actin filament reorganization. **a** Cultured rat hippocampal neurons stained using Alexa-Fluor-647-phalloidin in the absence or presence of oxygen and glucose deprivation (OGD). Selected inverted grayscale somatodendritic regions are highlighted in pink. Note: in control F-actin is concentrated in dendritic spines (dark puncta), while OGD induces a decrease in spine F-actin and an accumulation of linear bundles of F-actin within the soma and dendrite of many neurons (actinification). Scale bar, 5 µm. **b** Numbered insets correspond to blue boxed regions in **a**. Width blue boxes = 17 µm. **c** Quantification of the fraction of actinified neurons in control vs OGD or OD (oxygen deprivation) for the indicated durations; the NMDA receptor antagonist APV prevented actinification seen with 6 h OGD. *n* = 4 independent experiments; Kruskal–Wallis one-way ANOVA (*p* = 0.0066), with Dunn’s post hoc multiple comparison analysis (*p* = 0.0421 OGD 0 m vs 6 h; *p* = 0.0149 OGD 6 h vs APV). **d** Coronal brain section of a Thy1-YFP (green) expressing mouse stained for the neuronal cell body marker NeuN (magenta) 6 h post stroke. Regions marked “1” and “2”: areas from which the corresponding enlarged images in **e** are shown (below). Scale bar, 350 µm. Right: Higher magnification of the individual fluorescent labels of the ischemic area. **e** Alexa-Fluor-647-Phalloidin staining of neurons within somatosensory cortex within (2) infarcted region (middle, right panels at different magnification), or (1) corresponding region contralateral to the stroke (left single panel). Blue arrows: accumulation of linear actin bundles within the somatodendritic region of YFP-positive neurons. F-actin staining within the control, contralateral region (left) exhibits the punctate pattern of dendritic spines. Scale bars, 5 µm (left and middle*)*, 8 µm (right). **f** Quantification of the fraction of YFP-positive layer 5/6 neurons exhibiting actinification under the following conditions and regions: sham-operated mice (somatosensory cortex); or stroke-induced mice within the contralateral cortex, ipsilateral cortex, the ischemic core, or the penumbra.; *n* = 3 biologically independent animals each, sham vs stroke; one-way ANOVA (*p* = 0.0001), with Tukey’s post hoc multiple comparison analysis (*p* = 0.0001 sham vs core; *p* = 0.0004 core vs pen). All graphs: data presented as mean ± SEM. Source data are provided as source data file.

To investigate actin filament responses to stroke-like conditions we exposed cultured rat brain hippocampal neurons to oxygen and glucose deprivation (OGD). OGD is a well-established in vitro model for investigating neuronal cellular and subcellular responses to hypoxia and ischemia^17^. OGD induced a dramatic reorganization of F-actin within the soma and dendrites of neurons. Over 2–6 h, an increasing fraction of neurons showed a substantial loss of phalloidin staining for F-actin within dendritic spines, where F-actin is normally concentrated, and an aberrant accumulation of F-actin within the somatodendritic compartment (Fig. 1a–c). Filaments accumulated throughout the interior of the soma and proximal dendrites, and to various extents within more distal dendrites.

Because in most spiny neurons the soma and shaft of the dendrite is fairly devoid of F-actin relative to dendritic spines, we call this phenomenon the “actinification” of the neuron. OGD-induced somatodendritic actinification was completely blocked in the presence of the NMDA receptor antagonist (2 R)-amino-5-phosphonovaleric acid (APV; Fig. 1c). Interestingly, oxygen-deprivation alone was insufficient to induce actinification within the same time frame (Fig. 1c), perhaps because neurons sustain partial energy production via glycolysis for extended periods^18^. Together, these results suggest that actinification is induced by a catastrophic loss of ATP, leading to excess glutamate release and NMDA receptor hyperactivation.

### Actin reorganization induced after micro-infarct (stroke) in vivo

We investigated whether ischemia in vivo also induces neuronal actinification using either wildtype mice or transgenic Thy1 promoter-driven YFP expressing mice, the latter of which allowed us to readily identify dendritic arbor morphology in layer 2/3 and 5/6 cortical pyramidal neurons. Photothrombotic occlusion in single penetrating arterioles within the somatosensory cortex of mouse or rat brain induces small infarcts with well-defined borders^12,19^. Such strokes are greatly attenuated by application of NMDA antagonists before or immediately following blood vessel occlusion^12^. Within 2–6 h, the infarct region induced by unilateral single vessel photothrombosis was identifiable using a variety of markers, including fluorescent hypoxyprobe to detect severely hypoxic tissue, anti-immunoglobulin-G (anti-IgG) to detect vascular leakage, and reduced immunostaining for the neuron-specific proteins MAP2 and NeuN, as reported previously^20,21^ (Fig. 1d and Supplementary Fig. 1). Reductions in MAP2 have been attributed to proteolytic degradation by calpains in previous studies^22–24^. However, we noted that, consistent with a previous report^21^, the strongly reduced immunoreactivities for NeuN at this early time point reflected a loss of antibody staining, i.e., epitope loss, rather than a reduction in overall cell number, since DAPI staining for nuclear DNA and cytoplasmic YFP labeling were retained in neurons lacking NeuN (Supplementary Fig. 2). Actinification was detected within infarcts induced in either wildtype or Thy1-YFP mice.

Phalloidin staining revealed that in control brain tissue, as in control cultured neurons, the majority of F-actin was concentrated in dendritic spines (Fig. 1e and Supplementary Fig. 3). By 4–6 h after arteriole occlusion, the dendrites of YFP-expressing layer 5/6 pyramidal neurons showed a substantial loss or shrinkage of dendritic spines and the appearance of dystrophic dendrites, (Supplementary Fig. 4), similar to previous reports^15^. Low-magnification imaging of the infarct region indicated there was a time-dependent decrease in overall F-actin concentration, consistent with a loss of dendritic spine F-actin (Supplementary Fig. 5). However, high magnification imaging of individual layer 5/6 YFP-positive neurons showed aberrant accumulations of F-actin bundles within neuronal soma and proximal dendrites (Fig. 1e). Quantitative analysis revealed that within the ischemic core of the infarct (defined by the region of NeuN loss) there was a significant increase in the number of actinified neurons compared to control neurons defined in either sham treated mouse brains, in the stroke condition within contralateral cortex, or within ipsilateral temporal lobe cortex distant from the infarct region. Within the core of the infarct, nearly 90% of the YFP-labeled neurons showed actinification (Fig. 1f).

Dendritic spine shrinkage and spine F-actin loss have been described previously as an early response to stroke, OGD, or to NMDA/glutamate receptor hyperactivation, and in several cases actin-related mechanisms contributing to spine shrinkage were investigated^14–16,24–32^. However, the significant accumulation of F-actin seen in the somatodendritic compartment is unexplored, and is surprising given that F-actin polymerization typically involves ATP consumption. Logically speaking, actin polymerization would be disfavored in conditions of hypoxic cellular stress, when energy supplies become severely limited. We therefore focused on understanding the mechanism and biological relevance of somatodendritic actinification.

### NMDA receptor hyperactivation induces actinification within minutes

**Fig. 2:**
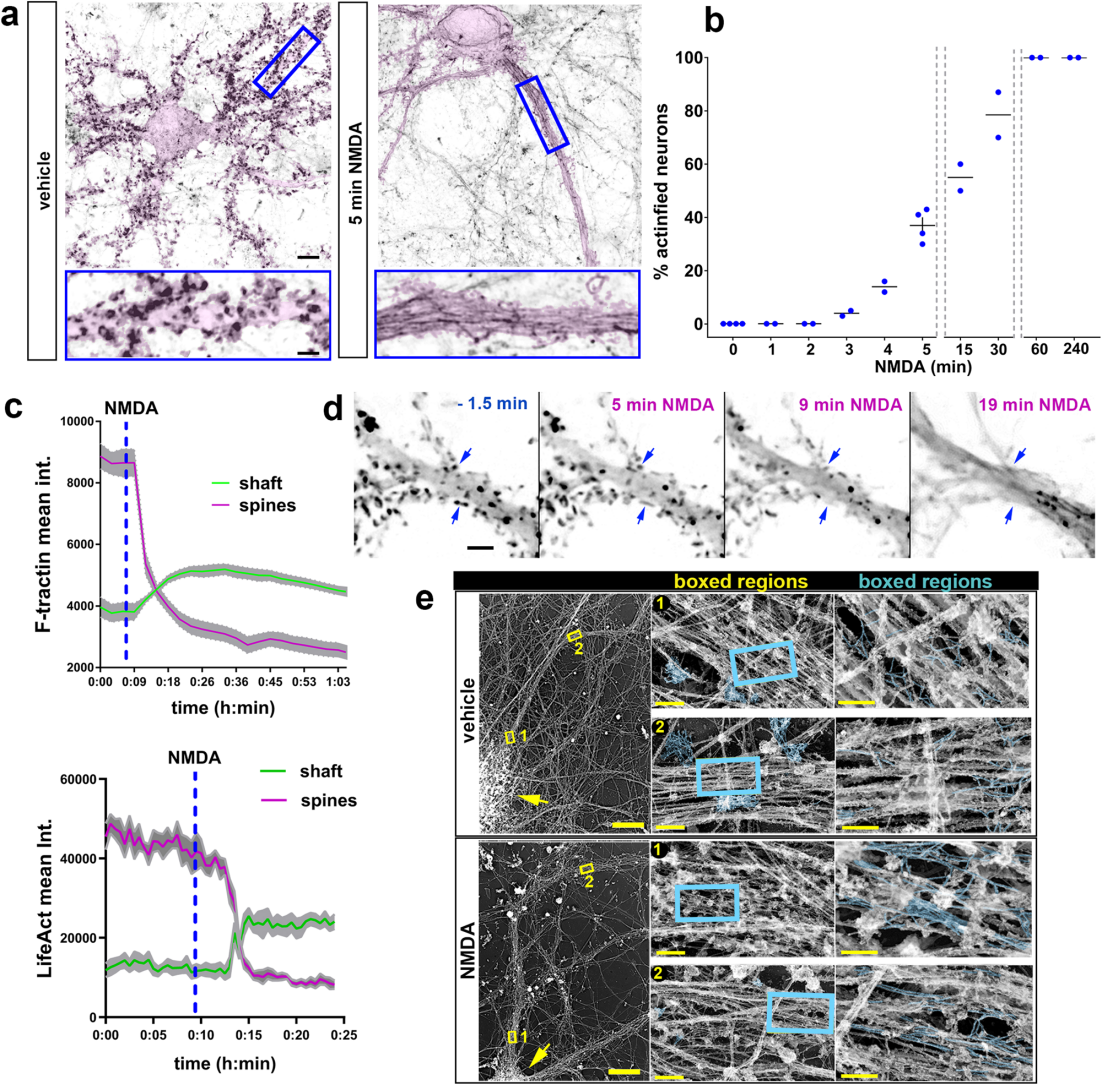
Rapid actinification induced by sub-lethal hyperactivation of NMDA receptors: time course and ultrastructure. **a** Neurons expressing membrane-tagged GFP, to emphasize morphology (pink highlight), and stained with Alexa-Fluor-647-phalloidin to monitor F-actin (reverse grayscale). Neurons fixed after 5 min incubation with vehicle (left) or with 50 μM NMDA (right). Insets below show enlarged regions boxed in. Scale bars, 8 µm (above); 2.8 µm (below). **b** Time course of actinification; *n* = 4 independent experiments for 0 and 5 min; *n* = 2 independent experiments for all the other time points. **c** Examples of two representative time courses where either mApple-F-tractin or Lifeact-mRFP intensity were monitored over time in an individual dendrite shaft (green line) vs the adjacent spines (magenta line) during NMDA-induced actinification. Plotted lines represent mean ± SEM from regions of interest (ROIs) taken across different portions of the same dendrite and different spines of a single neuron. **d** Time-lapse montage of a dendritic region expressing Lifeact-mRFP at selected time points before and after exposure to NMDA. Scale bar, 5 µm. Blue arrows: examples of spine F-actin decreasing as the F-actin signal in the shaft increases. **e** Hippocampal cultures incubated for 30 min in vehicle or NMDA, prior to processing for platinum replica electron microscopy (PREM). A neuron representative of each condition is shown at left; yellow arrows: cell soma; yellow boxes: two regions of the dendrite that are displayed at 70× higher magnification in the middle panels, and at a further 3× higher magnification in the right panels. Actin filaments are highlighted (cyan). In control (vehicle), numerous clusters of short, branched actin filaments are observed in presumptive dendritic spines, with occasional actin filaments within the dendrite shaft. After NMDA spine-like protrusions substantially decreased, and numerous long, unbranched filaments were detected within the dendritic shaft (cyan labeled structures within boxed regions). Scale bars, 20 µm for left panels, 500 nm for middle panels, and 200 nm for right panels. All graphs: data presented as mean ± SEM, and source data are provided as source data file.

Since an NMDA receptor antagonist completely prevented somatodendritic actinification following OGD, we asked whether actinification could be triggered via direct activation of NMDA receptors. Incubation of cultured hippocampal neurons with 50 µM NMDA induced a time-dependent increase in neuronal actinification, accompanied by extensive loss of dendritic spine F-actin and shrinkage of spines, similar to that seen following OGD in vitro and stroke in vivo (Fig. 2a). However, the time course for the actinification response was greatly accelerated. Based on a time series collected using fixed cultures, half-maximal actinification (the time at which 50% of all neurons were actinified) occurred after 5–10 min (average *t*_1/2_ = 7 min) in the sustained presence of NMDA, reaching nearly 100% by 60 min (Fig. 2b).

### Time-lapse imaging of actinification

We carried out time-lapse imaging of individual hippocampal pyramidal neurons using either Lifeact-mRFP (Supplementary Movie 1), mApple-F-tractin, or the small molecule dye far-red silicon-rhodamine actin (SiR-actin; Supplementary Movie 2). The time course of live neurons undergoing actinification was consistent with the time course determined using fixed populations of neurons. Time-lapse imaging of individual neurons revealed that timing of the actinification of the somatodendritic compartment closely overlapped with the decrease in dendritic spine F-actin. As with fixed samples, live imaging showed that most neurons underwent actinification after variable delays ranging from 3 to 20 min post-NMDA. However, once detectably initiated, actinification proceeded rapidly and with a similar time course (i.e., over the next ~4–6 min) in all neurons observed. The decrease in dendritic spine F-actin persisted during this period. Due to the variability in delay and speed, we could not readily average time curves from live experiments. Figure 2c shows two representative examples of individual neurons assayed in spines vs shaft for F-actin levels during live imaging (note that two different F-actin probes are shown). Figure 2d is a time montage image series taken at high magnification, illustrating the spatiotemporal correlation between spine F-actin loss and shaft F-actin accumulation.

### Ultrastructural analysis indicates that actinification results in long, unbranched actin filaments

The rapid time course of actinification initiated by NMDA suggests that the aberrant accumulation of F-actin filaments is likely due to an enzymatically driven process, rather than the slower aggregation type process that governs build-up of pathological aggregates in neurodegenerative diseases such as Alzheimer’s. To investigate the characteristics of actinified dendrites at the ultrastructural level, we carried out platinum replica electron microscopy (PREM) on control cultures and compared them to cultures incubated with 50 µM NMDA for 5 or 30 min (Fig. 2e). PREM images from control neurons revealed numerous clusters of short, moderately branched actin filaments localized within dendritic spine-like structures along the dendrite shaft, consistent with previous studies^33^. Within the dendrite shaft of control neurons, actin filaments were relatively sparse. Long actin filaments running parallel to the main dendrite axis were rarely observed, and when detected they usually exhibited occasional branches. In contrast, and in agreement with our light microscopy observations, NMDA-treated cultures revealed numerous instances of long, mostly unbranched filaments, which often formed irregular bundles. In parallel, there was a substantial decrease in the frequency of spine-like protrusions containing branched actin filaments that were common in control neurons. These observations suggest that dendrite actinification is driven by a process that disassembles branched actin filaments in spines and polymerizes F-actin into unbranched, rather than branched, filaments in dendrite shafts.

### Actin filaments induced by NMDA are extremely stable

**Fig. 3:**
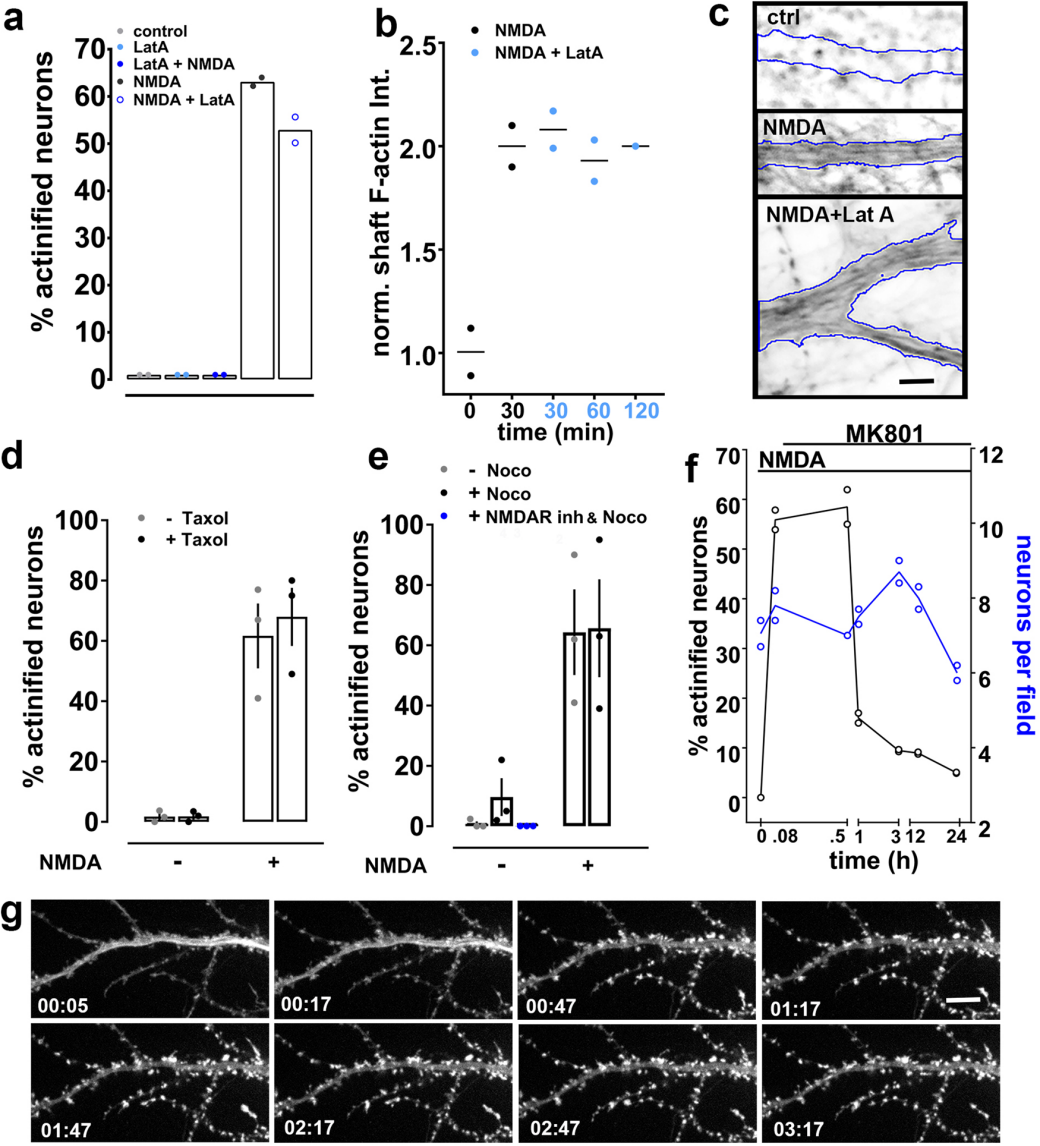
Actinification induces highly stable actin filaments that spontaneously disassemble upon stress removal. **a** Prior latrunculin A (LatA) prevents actinification (LatA + NMDA), but does not reverse actinification when added for up to 2 h after NMDA (NMDA + LatA). *n* = 2 independent experiments; Kruskal–Wallis one-way ANOVA (*p* = 0.0159). **b** Phalloidin intensity within proximal dendrites of neurons exposed to NMDA for 30 min with LatA for the indicated times. *n* = 2 independent experiments; Kruskal–Wallis one-way ANOVA (*p* = 0.3471). **c** Selected phalloidin-stained dendrites illustrating that NMDA-induced actinification persists after 2 h incubation with LatA (dendrite borders outlined in blue). Scale bar, 4 µm. **d** Taxol (10 nM, 30 min) neither prevents NMDA-induced actinification, nor induces actinification in absence of NMDA. *n* = 3 independent experiments; two-way ANOVA [(−)NMDA vs (+) NMDA, *F* (1, 8) = 75.82; *p* = 0.0001; (−)taxol vs (+) taxol, *F* (1, 8) = 0.1954, n.s. *p* = 0.6702]. **e** Nocodazole neither prevents NMDA-induced actinification nor induces it in the presence of an NMDAR antagonist cocktail (MK-801, APV, CGP). *n* = 3 independent experiments; two-way ANOVA [(−) NMDA vs (+) NMDA, *F* (1, 8) = 28.35; *p* = 0.0007; (−) nocodazole vs (+) nocodazole, *F* (1, 8) = 0.2064, n.s. *p* = 0.6616]. Mann–Whitney test, two-tailed [nocodazole(+) vs nocodazole + NMDAR antagonists (ANT), n.s. *p* = 0.1]. **f** Reversibility of actinification after the addition of the NMDA antagonist MK-801 (25 µM) following 5 min NMDA. Total number of neurons per same field (blue circles) quantified to establish that the decrease in actinified neurons was not due to cell death. *n* = 2 independent experiments. **g** Time-lapse images collected at the indicated times (hours: minutes) from an actinified dendrite expressing mApple-F-tractin to monitor live the F-actin reorganization following NMDA stress removal (at *t* = 0) via MK-801 addition. Note the clearing of the accumulated shaft F-actin, and the re-emergence of F-actin in spine-like protrusions. Scale bar, 8 µm. All graphs: data presented as mean ± SEM, and source data are provided as source data file.

Once formed, actin filaments in the actinified neuronal somata and dendrites appeared to be highly stable. We tested whether incubation with latrunculin A would accelerate the removal of the actinified F-actin in the dendrite shaft. Actin filaments within dendritic spines turnover with a half-time of <1 min^34,35^. We reasoned that if a similarly high turnover rate of F-actin characterizes the actinified dendritic compartment, we should observe that application of the G-actin sequestering compound Latrunculin A—applied after actinification has occurred—would greatly speed the net disassembly of the filaments. However, our observations did not support this hypothesis. Incubation with 2 µM LatA for up to 2 h after actinification induction caused no detectable decrease in the percentages of actinified neurons, nor loss of F-actin staining intensity within individual neurons in the soma and dendrites (Fig. 3a–c). Note that we first confirmed that LatA would prevent actinification, as expected, when applied before actinification was induced by NMDA (Fig. 3a), consistent with actinification being an actin monomer-dependent polymerization process. The resistance of filaments to latrunculin added post-actinification suggests that the newly polymerized F-actin induced by NMDA is extremely stable in the continued presence of NMDA.

### Actinification is independent of microtubule stability

We examined whether manipulations of microtubule stability would alter the induction of actinification. Taxol had no effect on the propensity of neurons to actinify under control conditions (Fig. 3d); however, we observed a tendency for nocodazole to induce actinification of a small number of neurons under control conditions, but this effect was completely prevented in the presence of NMDA antagonists, indicating that nocodazole may slightly increase spontaneous glutamate release (Fig. 3e). Incubation with neither the microtubule stabilizing compound taxol (Fig. 3d) nor the microtubule polymerization inhibitor nocodazole prevented NMDA-induced actinification (Fig. 3e), indicating that stable microtubules are not essential to actinification.

### Spontaneous reversal of actinification upon stress removal

Despite the apparently non-dynamic nature of the F-actin induced by NMDA, the actinification filaments spontaneously disassembled upon removal of NMDA. When cultures were incubated with NMDA for 5 min, followed by addition of MK-801 to prevent further NMDA receptor activation, the fraction of neurons in fixed cultures showing actinification dropped from about 60% to <20% within 1 h (Fig. 3f). By 3 h, only 8% of neurons remained actinified, and by 12 h only 4% of neurons remained actinified. Over this same time frame, there was no significant decrease in the number of live neurons, although there was a trend toward decreased neuronal viability by 24 h (Fig. 3f). On average, the time for half-maximal recovery after a 5 min incubation with NMDA was approximately 30 min.

Time lapse imaging of individual neurons similarly documented that actinification was reversible, and that F-actin within the somatodendritic compartment spontaneously returned to a control-like distribution following arrest of the ongoing NMDA stress. Figure 3e shows a neuron expressing mApple-F-tractin, illustrating that the NMDA-induced accumulation of F-actin began to detectably clear from the dendrite shaft within 17 min of the addition of NMDA antagonists. Simultaneously, F-actin re-emerged in dendritic spines, thus returning to a qualitatively normal distribution within 1 h (Fig. 3e). These observations indicate that actinification persists only while the NMDA receptor hyperactivation persists, and that endogenous mechanisms support the spontaneous depolymerization of F-actin in the dendrite shaft/soma and the re-polymerization of F-actin in dendritic spines.

### Actinification is triggered by conditions that elicit neuronal swelling

**Fig. 4:**
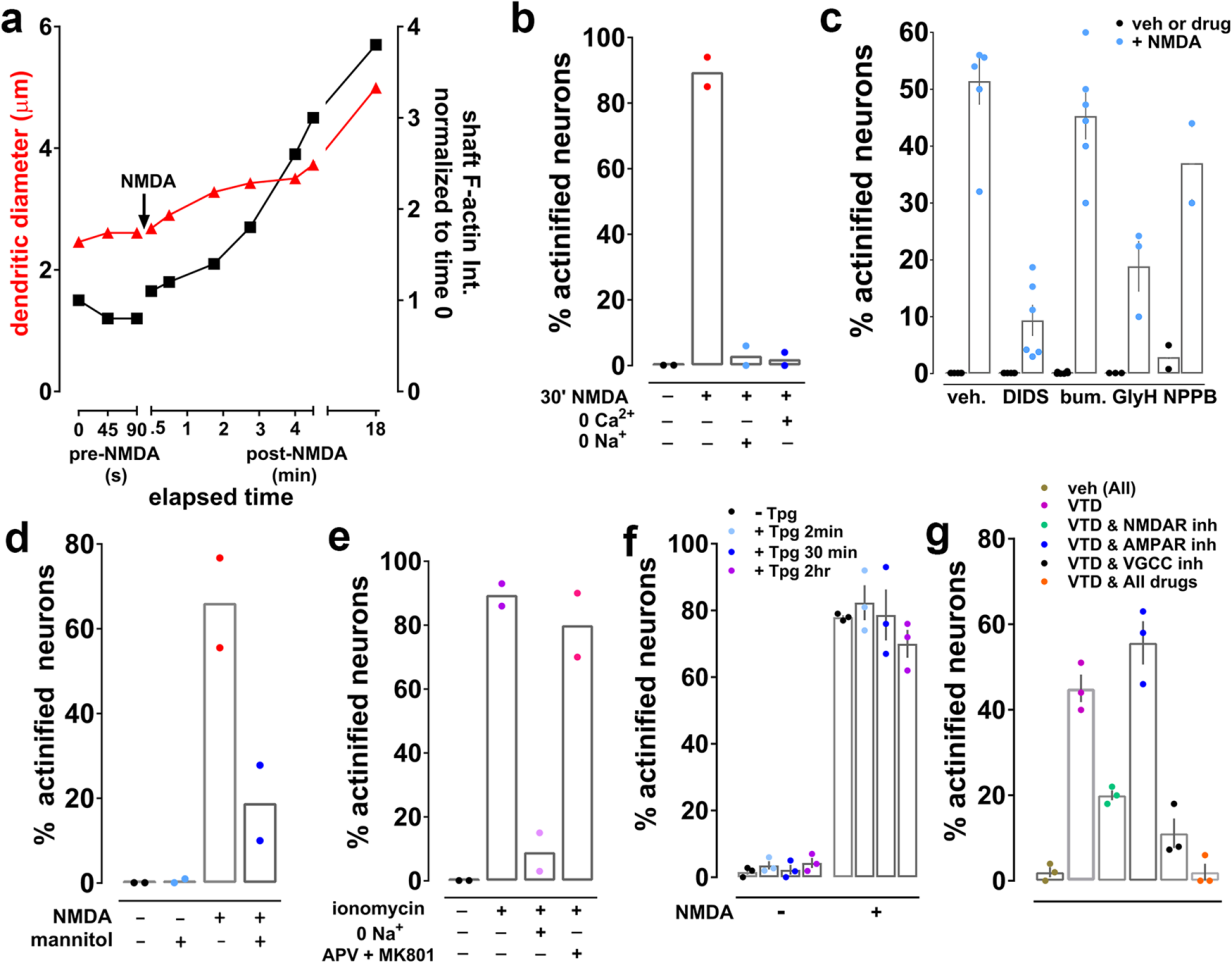
Actinification is triggered during cell swelling. **a** Simultaneous quantification of dendrite diameter (red triangles) and F-actin intensity (black squares) in an individual neuron following addition of NMDA at the indicated time. **b** Removal of either extracellular sodium or calcium prevents NMDA-induced actinification. *n* = 2 independent experiments; unpaired *t* test, two tailed (*p* = 0.0039, NMDA vs NMDA w/ zero Na^+^; *p* = 0.0032, NMDA vs NMDA w/ zero Ca^2+^). **c** Effect of various chloride flux inhibitors on NMDA-induced actinification. *n* = 5 (veh; DIDS; bum); 3 (GlyH); 2 (NPPB) independent experiments. Two-way ANOVA [(−) NMDA vs (+) NMDA, *F* (1, 36) = 230.8; *p* < 0.0001; all drugs, *F* (4, 36) = 0.1954, *p* < 0.0001]. **d** Mannitol inhibits NMDA-induced actinification. *n* = 2 independent experiments; two-way ANOVA [(−)NMDA vs (+) NMDA, *F* (1, 4) = 11.85; *p* = 0.0262; (−) mannitol vs (+) mannitol, *F* (1, 4) = 11.41, *p* = 0.0279], with Tukey’s post hoc multiple comparison analysis (*p* = 0.0285 NMDA w/ mannitol vs NMDA w/o mannitol). **e** Ionomycin induced actinification in the absence (−) or presence (+) of either extracellular sodium depletion or a cocktail of the NMDAR antagonists APV and MK-801. *n* = 2 independent experiments; Kruskal–Wallis one-way ANOVA (*p* = 0.0286); unpaired t-test (*p* = 0.0074, iono vs iono w/ zero Na^+^; n.s. *p* = 0.4645, iono vs iono w/APV &MK801). **f** Thapsigargin (Tpg) neither induces or prevents NMDA-induced actinification. *n* = 3 independent experiments; two-way ANOVA [(−)NMDA vs (+) NMDA, *F* (1, 16) = 805.1; *p* = 0.0001; Tpg (2 min), vs (30 min), vs (2 h), *F* (3, 16) = 0.8284, n.s. *p* = 0.4974]. **g** Veratridine (VTD) induces actinification by activating NMDARs and voltage gated calcium channels (VGCCs), but not AMPARs. Three cocktails of inhibitors were used against either NMDARs (APV, MK-801, CGP), AMPARs (NBQX), or VGCCs (nifedipine, ω-conotoxin, mibefradil); respective vehicles were tested in combination. *n* = 3 independent experiments; Kruskal–Wallis one-way ANOVA (*p* = 0.0071); unpaired *t* test, two-tailed (*p* = 0.0019, VTD vs VTD + NMDAR antagonists (ANT); n.s. *p* = 0.1491, VTD vs VTD + AMPAR ANT; *p* = 0.002, VTD vs VTD + VGCCs inhibitors (INH); *p* = 0.0003 VTD vs VTD + All drugs). All graphs: data presented as mean ± SEM, and source data are provided as source data file.

Most of the early neuronal cell death induced during ischemia occurs via pathological cell swelling (also called cytotoxic edema)^36,37^. We observed that somatodendritic actinification occurred in parallel with swelling of the cell body and adjacent dendrite (an example from an individual neuron is shown in Fig. 4a; as described above, variable delays in the initiation of actinification preclude averaging the time course data). We therefore asked whether actinification was provoked by the same conditions that cause osmotic cell swelling, namely influx of both sodium and chloride, followed by water. First, we observed that replacement of extracellular sodium chloride by *N*-methyl-d-glucamine chloride completely prevented NMDA-induced actinification (Fig. 4d), consistent with the hypothesis that sodium flux across the plasma membrane is critical for actinification. Recent studies demonstrated that voltage-dependent chloride influx through the solute carrier family 26 member 11 (SLC26A11) ion exchanger drives neuronal Cl^−^ entry during glutamate-induced neuronal cell swelling^10^. We found that actinification exhibited a similar pharmacological profile for chloride influx inhibitors as that reported for cytotoxic edema in brain tissue^10^. 4,4′-Diisothiocyanatostilbene-2,2′-disulfonic acid (DIDS) and glycine hydrazide (GlyH-101), which inhibit Cl^−^ entry via SLC26A11^10^, significantly inhibited actinification, but neither bumetanide, which blocks the brain Na-K-Cl cotransporter 1 (NKCC1) cation-chloride cotransporter, nor 5-nitro-2-(3-phenylpropylamino) benzoic acid (NPPB), which blocks volume regulated Cl^−^ channels and Ca^2+^-activated Cl^−^ channels, were effective in preventing actinification (Fig. 4b). This indicates that actinification might require chloride entry via the same routes as reported for neuronal cell swelling.

We next tested whether water entry was necessary for induction of actinification. Incubation of cultures with the non-cell permeable sugar mannitol, to reduce the osmotic driving force for water entry, indicated that swelling was indeed contributing to NMDA-induced actinification (Fig. 4c).

### Calcium entry is necessary but not sufficient for actinification

NMDA receptors are highly permeable to calcium ions, and increased intracellular calcium is a trigger for many biochemical cascades elicited by NMDA receptor activation. Removal of extracellular calcium completely prevented neuronal actinification (Fig. 4d). These data indicate that calcium influx across the plasma membrane is required to trigger actinification. However, calcium flux alone was insufficient, because although 10 min incubation with the calcium ionophore ionomycin induced substantial actinification, even in the presence of NMDA receptor antagonists, this effect was completely blocked when extracellular sodium was removed (Fig. 4e). Collectively, our results indicate that sodium, chloride, and calcium influx are all required to induce actinification, and that water entry and consequent neuronal swelling is also a critical factor.

We next asked whether calcium released from intracellular endoplasmic reticulum (ER) stores participates in actinificiation in neuronal cultures. However, we found that thapsigargin, an inhibitor of the ER Ca2+ ATPase, neither stimulated actinification on its own nor inhibited NMDA-induced actinification (Fig. 4f), indicating that release of ER calcium stores is neither sufficient nor necessary to induce actinification.

Finally, we investigated whether, in addition to NMDA receptors, alternative routes of calcium and sodium entry might trigger actinification. We incubated cultures with veratradine, a blocker of voltage-sensitive sodium channel inactivation that induces neuronal depolarization^38^, and examined its effect in the absence and presence of antagonists of NMDA receptors, AMPA receptors, and/or voltage-gated calcium channels (VGCC; Fig. 4g). Veratridine was indeed able to induce actinification in ~40% of neurons, and this level was reduced to 20% in the presence of an NMDA antagonist cocktail. In contrast, AMPA receptor antagonism had no effect in reducing the effect of veratridine. On the other hand, the effect of veratridine was strongly reduced, but not completely prevented, in the presence of VGCC inhibitors. However, a cocktail of inhibitors that collectively block NMDA receptors, AMPA receptors, and VGCCs completely prevented veratridine-induced actinification (Fig. 4g). Taken together, these veratridine experiments indicate that, although ionic imbalance induced by NMDA receptor hyperactivation strongly drives actinification via sodium and calcium entry, actinification can also be induced in the absence of NMDA receptor activity when neuronal depolarization permits calcium entry through voltage-gated calcium channels.

Overall, the requirement for sodium, chloride, and water entry together with calcium entry from extracellular sources suggests that actinification may be optimally be triggered by the convergence of both cell swelling and calcium influx.

### Inverted formin 2 is required for actinification

**Fig. 5:**
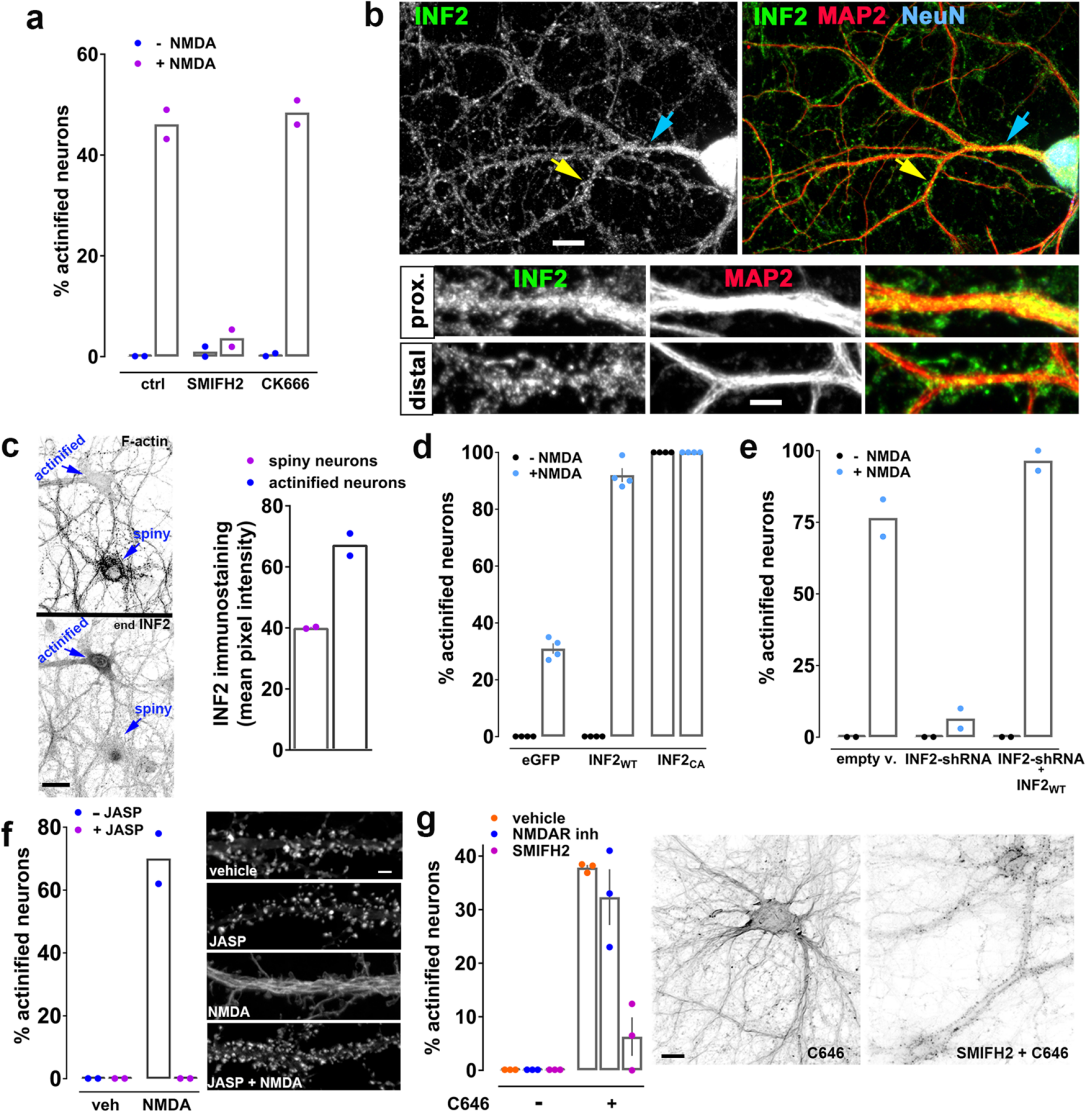
Actinification requires INF2 and F-actin depolymerization. **a** SMIFH2, not CK666, inhibits actinification. *n* = 2 independent experiments; two-way ANOVA [(−) NMDA vs (+) NMDA, *F* (1, 6) = 513.2; *p* = 0.0001; ctrl vs SMIFH2 vs CK666, *F* (2, 6) = 101, *p* = 0.0001], with Tukey’s post hoc multiple comparison analysis (*p* < 0.0001 NMDA vs NMDA w/ SMIFH2; n.s. *p* = 0.9168 NMDA vs NMDA w/ CK666). **b** INF2 immunoreactivity in neurons (NeuN). Arrows: distal (yellow) and proximal (blue) dendrites (MAP2), enlarged in lower panels. Scale bar, 12 µm (upper panels), 5 µm (lower panels). **c** INF2 immunoreactivity is higher in early-actinified than non-actinified neurons. Blue arrows: neuronal soma. Scale bar: 20 µm. *n* = 2 independent experiments; unpaired t-test,two-tailed (*p* = 0.0175). **d** Wildtype INF2 (INF2_WT_) enhances NMDA-induced actinification; constitutively active INF2 (INF2_CA_) actinifies without NMDA. *n* = 4 independent experiments; two-way ANOVA [(−) NMDA vs (+) NMDA, *F* (1, 18) = 1645; *p* = 0.0001; eGFP vs INF2_WT_ vs INF2_CA_, *F* (2, 18) = 2394, *p* = 0.0001], with Tukey’s post hoc multiple comparison analysis (*p* = 0.0001 eGFP +/− NMDA; *p* = 0.0001 INF2_WT_ +/− NMDA; n.s. p = 0.999 INF2_CA_ +/− NMDA). **e** INF2 shRNA prevents actinification; rescue by shRNA-resistant INF2_WT_. *n* = 2 independent experiments; two-way ANOVA [(−) NMDA vs (+) NMDA, *F* (1, 6) = 481.1; *p* = 0.0001; empty v. vs INF2shRNA vs INF2shRNA + INF2_WT_, *F* (2, 6) = 100.6, *p* = 0.0001], with Tukey’s post hoc multiple comparison analysis (*p* = 0.0001 empty v. +/− NMDA; n.s. *p* = 0.7496 INF2shRNA +/− NMDA; *p* = 0.0001 INF2shRNA & INF2_WT_ +/− NMDA). **f** Jasplakinolide (JASP) prevents NMDA-induced actinification (GFP-F-tractin). *n* = 2 independent experiments; two-way ANOVA [(−) NMDA vs (+) NMDA, *F* (1, 4) = 76.34; *p* = 0.0009; (−) JASP vs (+) JASP, *F* (1, 4) = 76.34; *p* = 0.0009]. Scale bar, 4 µm. **g** C646 formin-dependent actinification, despite NMDAR antagonists (APV + MK801). *n* = 3 independent experiments; two-way ANOVA [(−)C646 vs (+) C646, *F* (1, 12) = 144.4; *p* = 0.0001; vehicle vs NMDA antagonists vs SMIFH2, *F* (2, 12) = 21.2; *p* = 0.0001], with Tukey’s post hoc multiple comparison analysis (*p* < 0.0001 vehicle +/− C646; (*p* < 0.0001 NMDAR anta +/− C646; n.s. *p* = 0.5596 SMIFH2 +/− C646). Scale bar, 12 µm. All data are mean ± SEM. Source data are provided as source data file.

We next turned to identifying the key actin mechanisms involved in catalyzing neuronal actinification. The polymerization of most actin filaments in cells are initiated via two distinct mechanisms. The Arp2/3 complex nucleates and elongates daughter filaments from the side of an existing actin filament, thereby forming branched F-actin networks, as seen in lamellipodia and dendritic spines^39–41^. Conversely, formin-driven F-actin polymerization induces formation of unbranched actin filaments, as seen in filopodia and other structures where straight filaments predominate^39,42,43^. We applied either SMIFH2 or CK666, small molecule inhibitors of formin-mediated and Arp2/3-mediated actin polymerization, respectively. Only the formin inhibitor prevented NMDA-induced actinification (Fig. 5a). This result is consistent with the ultrastructural observation that NMDA induced long, unbranched actin filaments (Fig. 2d).

SMIFH2 was recently reported to inhibit certain classes of myosins in addition to formins^44^. Therefore, we performed additional experiments to ascertain whether SMIFH2 might prevent NMDA-induced actinification through myosin inhibition. Myosins II and V are among the myosins inhibited by SMIFH2^44^, and are known to be present and functional in mature neuronal dendrites^45,46^. Pre-incubation of cultured neurons with selective inhibitors of each of these myosin family members had no effect in preventing NMDA-induced actinification (Supplementary Fig. 6), supporting the conclusion that SMIFH2 most likely acts via formin inhibition to prevent actinification.

Formins constitute a large superfamily of molecules, with fifteen mammalian formin genes identified to date^47^. Our attention was drawn in particular to inverted formin 2 (INF2), a member of the diaphanous subclass of formins. We found that immunoreactivity for INF2 is present throughout the somatodendritic domain of cultured hippocampal neurons, and distributes in a punctate fashion within both proximal and distal dendrites (Fig. 5b). No such staining was observed when endogenous INF2 was depleted from individual neurons via RNA interference (Supplementary Fig. 7).

Interestingly, we observed variability in the relative concentrations of INF2 immunoreactivity across control neurons within the same culture. We therefore treated cultures for 5 min with NMDA (a time when typically, 35–60% the neurons have become actinified) and quantified the level of immunoreactive staining for INF2 in the soma of actinified neurons vs non-actinified neurons (Fig. 5c). We observed a significantly higher degree of INF2 staining in the neurons that were the “early responders”—i.e., those that underwent actinification at this early time point. As 5 min is not likely to be sufficient time to alter the concentration of INF2 via the induction of protein synthesis (and we additionally confirmed that the protein synthesis inhibitor cycloheximide did not prevent NMDA-induced actinification; Supplementary Fig. 8), this bias in endogenous INF2 level implies that the pre-existing concentration of INF2 may partly determine the strength or timing of the actinification response in individual neurons.

To test this hypothesis directly, we transfected neurons with a constitutively active form of INF2 and observed that neuronal soma and dendrites showed actinification even in the absence of NMDA exposure (Fig. 5d). More importantly, neurons transfected to ectopically express a fluorescently tagged wildtype form of INF2 (GFP-INF2-wt) showed no increase in actinification in the absence of NMDA, but showed a dramatically enhanced actinification response when stimulated with NMDA for 5 min, with nearly all the neurons becoming actinified within this short time frame (Fig. 5d).

Conversely, silencing of endogenous INF2 expression using an shRNA completely blocked the actinification of the neuron, an effect that was rescued in the presence of an shRNA-resistant form of wildtype INF2 (Fig. 5e). Both CAAX and non-CAAX variants of INF2-wt^48^ were tested, and as we observed no statistical difference in the degree of rescue between them we combined the data into a single group. Together, these observations implicate INF2 as a critical driver of NMDA-induced actinification. They also indicate that INF2 activity normally remains low under control conditions regardless of INF2 concentration, and must be induced by a stimulus, since overexpression of wildtype INF2 had no detectable effect on actinification under resting conditions.

### A role for elevated G-actin, but perhaps not for deacetylases, in NMDA-induced actinfication

Recent studies showed that INF2 is maintained in an inactive conformation in cells^49^ and is then activated by stimuli that raise intracellular calcium^50–52^. One mechanism for this activation might be an increase in G-actin levels, since INF2 is known to be stimulated by elevated actin monomer concentrations^49^. We therefore hypothesized that the G-actin released during NMDA-induced depolymerization of spine F-actin might activate INF2. In support of this hypothesis, adding jasplakinolide during the 5 min NMDA incubation completely prevented both spine F-actin disassembly and dendritic actinification (Fig. 5f).

INF2 has also been proposed to be negatively regulated by binding to a complex consisting of cyclase-associated protein (CAP) and acetylated-G-actin. This complex is disrupted when G-actin becomes deacetylated, thereby allowing activation of INF2^53,54^. We observed that incubation of cultured neurons with the acetyltransferase inhibitor C646 strongly induced actinification, an effect that was not blocked by NMDA antagonists, consistent with a role for acetylation in regulating actinification (Fig. 5g). Notably, the C646-induced actinification was almost completely blocked by preincubation with the formin inhibitor SMIFH2 (Fig. 5g), similar to actinification induced by NMDA (Fig. 5a), suggesting they are mediated by the same INF2-dependent mechanism. We therefore hypothesized that INF2 activity might become induced in response to NMDA via the activation of cytosolic deacetylases (which are typically called histone deacetylases, or HDACs, even though it is now known that they can deacetylate numerous cytosolic substrates). As shown in Table 1, pre-incubation with several inhibitors of Class 1 or Class 2 HDACs modestly reduced NMDA-induced actinification, but none were robustly effective, including the HDAC6 inhibitor tubastatin, which blocked INF2 activity in non-neuronal cells^54^. Moreover, combinations of multiple inhibitors qualitatively showed little or no additive effect toward inhibiting actinification. Therefore, while our data suggest that de-acetylation is a factor in regulating neuronal actinification, the precise mechanisms that lead to INF2 activation in response to NMDA-induced cellular edema might not require deacetylase activity.

**Table 1:** HDAC inhibitors (HDI) and their efficacy in reducing NMDA-induced actinification

HDAC inhibited	Treatment (1 h HDI + 5 min NMDA)	% Reduction in actinification (*n*)
Class I + II	SAHA (1 µM)	40 ± 6 (5)
Class I	Santacruzamate (0.3 µM)	33 ± 3 (3)
Class I	CI994 (1 µM)	30 ± 4 (3)
Class I	FK228 (1 µM)	36 ± 3 (6)
Class II	Tubastatin (5 µM)	21 ± 6 (6)
Class II	Tubacin (10 µM)	n.d.q.
Class III	Nicotinamide (2 mM)	n.d.q.
Class III	Ex527 (2.5, 5, 30, 100 µM)	n.d.q.
Class III	anti-SRT5 peptides (20 µM)	n.d.q.
Class III	Suramin (100 µM)	n.d.q.

Cultured hippocampal neurons were incubated for 1h with HDAC inhibitors (HDI) before incubating the cells for 5 min with 50 µM NMDA. Concentrations below and above the ones listed were also tested to reach either no or toxic effect. n.d.q. = no inhibition detected in qualitative analyses; *n* = number of independently repeated experiments.

### NMDA-induced accumulations of actin in the shaft are distinct from cofilin-actin rods

Previous studies have reported that excess neuronal glutamate or hypoxic stress induce the accumulation of aberrant F-actin containing structures called cofilin-actin rods, which are defined by the concentrated presence of the actin severing protein ADF/cofilin along bundles of F-actin^55^. Such actin rods are not detectable by phalloidin staining, since the prominent binding of cofilin prevents the binding of phalloidin in such filaments^56^. Four lines of evidence indicate that the actinification of the dendrite compartment is a different process than the formation of cofilin-actin rods. First, unlike cofilin-actin rods, the actin filaments we observe are clearly labeled by phalloidin. Second, these filaments do not contain high concentrations of cofilin immunoreactivity (Supplementary Fig. 9a). Third, the spatiotemporal dynamics of actinification and the emergence of cofilin-actin rods differ qualitatively. Cofilin-actin rods reportedly appear in dendrites only after 30 min or more of continuous exposure to glutamate or NMDA^55^. Although we can detect cofilin-actin rods after a lengthy exposure of our cultures to glutamate (Supplementary Fig. 9b, c), typically they appear mainly in the distal dendrites and along axons surrounding the dendritic arbor. In contrast, actinification occurs within less than 5 min and is preferentially detected in the cell body and the proximal dendrites. Finally, even cofilin rods induced without NMDA by ectopic expression of wild type cofilin together with one of its activating phosphatases, chronophin, do not promote neuronal actinification (Supplementary Fig. 9d). Together, these observations strongly argue that somatodendritic actinification is a process that is distinct from cofilin-actin rod formation.

### Actinification is a pro-survival response

**Fig. 6:**
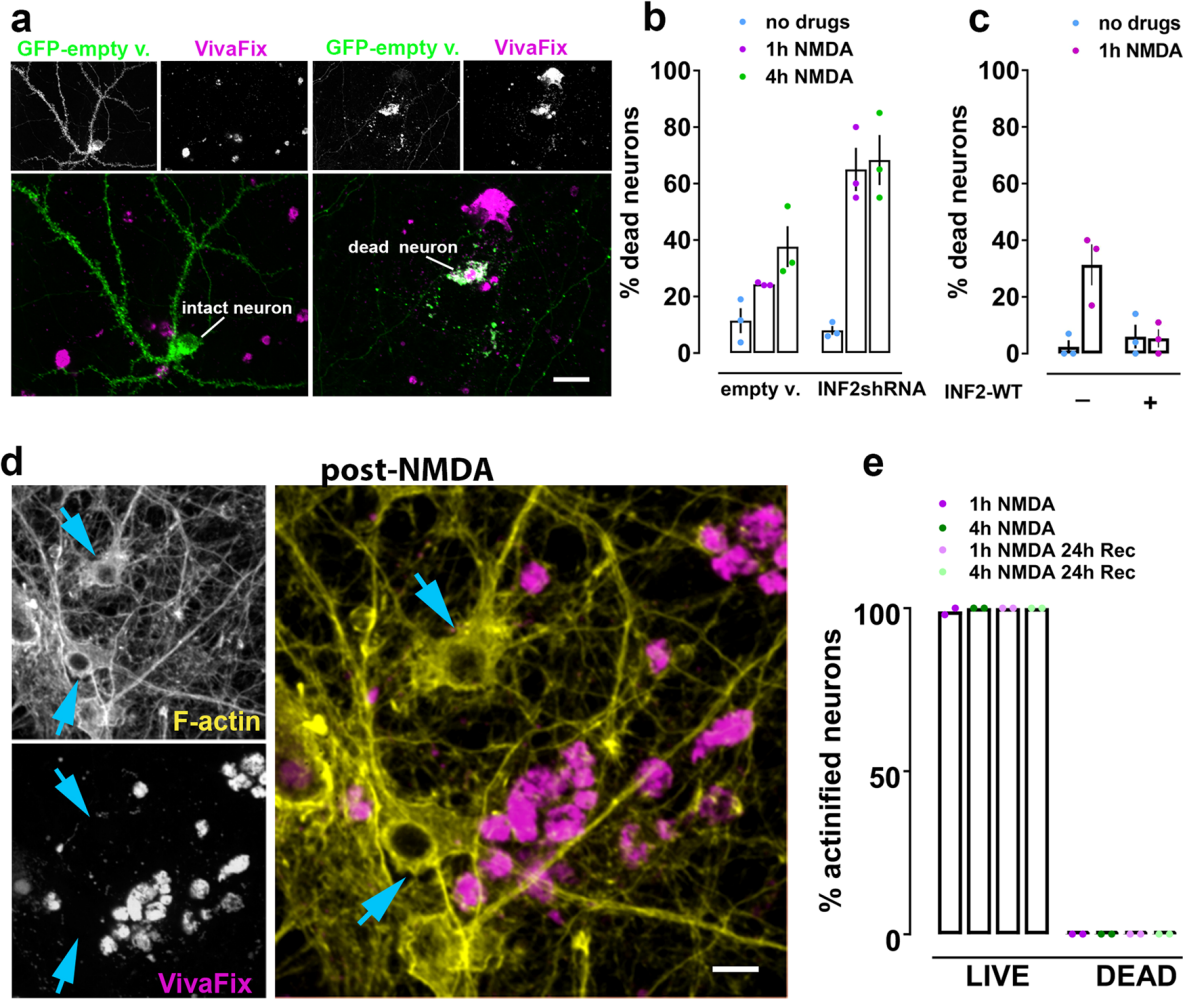
INF2 silencing enhances NMDA-induced neuronal cell death. Neurons expressing either control empty vector or its corresponding INF2-shRNA were incubated with or without NMDA prior to fixation and co-staining with Alexa-fluor-568-phalloidin and VivaFix 649/660. **a** Representative images of dead neurons (incorporating VivaFix) vs live neurons (excluding VivaFix, magenta). GFP-empty vector (green) was used as cytoplasmic filler to evaluate integrity of neuronal morphology (i.e., intact vs fragmented). Scale bars, 28 µm. **b** INF2 silencing increases cell death. *n* = 3 independent experiments; two-way ANOVA [empty v. vs INF2shRNA, *F* (1, 12) = 21.94; *p* = 0.0005; +/− NMDA (1 h, 4 h), *F* (2, 12) = 30.12; *p* = 0.0001], with Tukey’s post hoc multiple comparison analysis (n.s. *p* = 0.998, no drugs +/− INF2shRNA; *p* = 0.004, 1 h NMDA +/− INF2shRNA; *p* = 0.0298, 4 h NMDA +/− INF2shRNA). **c** Over-expression of wildtype INF2 (GFP-INF2WT) prevents NMDA-induced cell death. *n* = 3 independent experiments; two-way ANOVA [+/− INF2WT, *F* (1, 8) = 5.868; *p* = 0.0417; +/− NMDA (1 h), *F* (1, 8) = 9.444; *p* = 0.0153], with Tukey’s post hoc multiple comparison analysis (*p* = 0.0092, no INF2WT +/− NMDA; n.s. *p* = 0.9996, INF2WT +/− NMDA). **d** Neurons incubated for 1 h with NMDA and fixed immediately, illustrating that actinified neurons (yellow = phalloidin) do not take up VivaFix (magenta). Scale bars, 14 µm. **e** Quantification of live vs dead fraction of actinified neurons among neurons transfected with empty vector revealed a binary fate for stressed neurons. Actinified neurons always excluded VivaFix dye; neurons taking up VivaFix dye were never actinified, whether fixed immediately after the indicated NMDA incubation time, or after an “imposed recover period” where a cocktail of NMDAR blockers was added to prevent ongoing excitotoxicity. *n* = 2 independent experiments; two-way ANOVA [Live vs Dead, *F* (1, 8) = 159041; *p* = 0.0001; NMDA conditions, *F* (3, 8) = 0.88; n.s. *p* = 0.4911]. All graphs: data presented as mean ± SEM, and source data are provided as source data file.

We next investigated the functional impact of INF2-dependent actinification. Because the distribution of actin filaments spontaneously returned to control values after cessation of the stressful stimulus, we reasoned that actinification might be a pro-survival response. To test this hypothesis, we incubated cultures with NMDA for 1 or 4 h and compared neuronal survival using the VivaFix cell viability assay in neurons that were either transfected with shRNA against INF2 or with an empty vector (Fig. 6b). For both conditions, neurons were co-transfected with eGFP to identify transfected cells, and we quantified the percentage of transfected neurons that took up the VivaFix dye as an indicator of cell death (Fig. 6a). We observed that after either a 1 or 4 h incubation with NMDA the prior silencing of INF2 approximately doubled the fraction of non-viable neurons.

In a second series of experiments we asked whether overexpression of INF2 would reduce NMDA-induced cell death, since we earlier showed (Fig. 5d) that overexpression of INF2 greatly enhances actinification. Indeed, such ectopic expression of INF2-wt significantly prevented actinification, in agreement with the hypothesis that actinification is pro-survival (Fig. 6c). Together with the above shRNA results, these data are indicative of a concentration-dependent effect of INF2 in neuroprotection.

Although neuronal cultures can recover from brief exposures to bath applied NMDA, continuous exposure of neurons to NMDA for an hour or more is highly excitotoxic, and known to induce extensive neuronal death over the ensuing 1–2 days^57^. Indeed, when we incubated cultures with NMDA for 1–4 h and fixed them after 24 h, we observed, as expected, that the vast majority of neurons had died. However, a very small number of live neurons were still reliably detected (i.e., they excluded the VivaFix dye), even after this strong excitotoxic stimulus. Remarkably, 100% of these late-surviving neurons displayed actinification (Fig. 6d, e). Conversely, none of the neurons that were identified as dead showed actinification (Fig. 6d, e) and, indeed, phalloidin staining was depleted in dead neurons.

### Formin inhibition exacerbates cell death after stroke in vivo

**Fig. 7:**
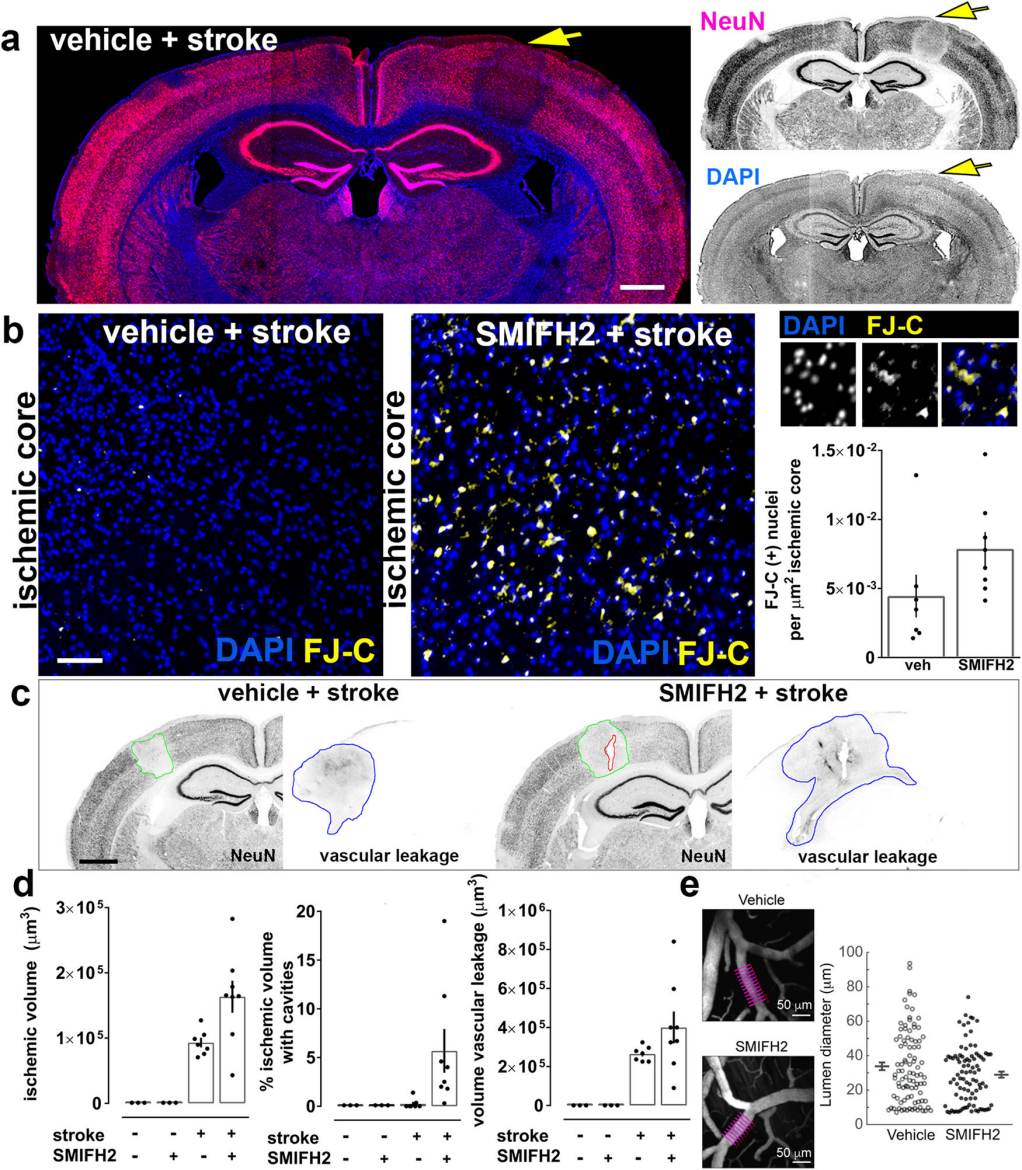
Formin inhibition enhances stroke-induced cell death in vivo. **a** Coronal mouse brain section showing reduced NeuN immunoreactivity within the infarct region (yellow arrow) 4 h post-stroke. Scale bar, 850 µm. **b** SMIFH2 increases early cell death as shown in representative regions of cortical ischemic core. Enlarged panels at right illustrate somatic FluoroJade-C (FJ-C) colocalization with nuclear DAPI staining. Quantification of the density of FJ-C-positive (+) soma within the ischemic core region. *n* = 7 animals (veh + stroke); *n* = 8 animals (SMIFH2 + stroke); Mann–Whitney test, two-tailed *p* = 0.0401; Scale bar: 70 µm. **c** Images of brain sections stained for the neuronal marker NeuN and for IgG to assess vascular leakage following stroke. Scale bars, 800 µm. **d** Quantification of infarct volume (based on NeuN signal loss), fraction of ischemic volume containing cavitations (holes in the brain parenchema), and vascular leakage (based on volume of IgG signal). *n* = 3 animals (veh./no stroke), *n* = 3 animals (SMIFH2/no stroke), *n* = 7 animals (veh + stroke), *n* = 8 animals (SMIFH2 + stroke). Ischemic volume: Kruskal–Wallis one-way ANOVA (*p* = 0.0015), with Dunn’s post hoc multiple comparison analysis (*p* = 0.0105 veh no stroke vs SMIFH2 stroke; *p* = 0.0105 SMIFH2 no stroke vs SMIFH2 stroke; n.s. vehicle stroke vs SMIFH2 stroke). Percentage of ischemic volume with cavities: Kruskal–Wallis one-way ANOVA (*p* = 0.0027). Volume vascular leakage: Kruskal–Wallis one-way ANOVA (*p* = 0.0005), with Dunn’s post hoc multiple comparison analysis (*p* = 0.0152 veh no stroke vs SMIFH2 stroke). **e** (Left) Representative in vivo two-photon imaging of cortical pial vessels from vehicle-treated (upper) and SMIFH2-treated (lower) mice. Purple lines denote the position of diameter measurements of a pial arteriole, averaged to provide a single data point. Scale bars, 50 µm. (Right) Scatter plot of pial arteriole diameters from vehicle and SMIFH2-treated groups, including 93 arterioles over 6 vehicle-treated mice, and 90 arterioles over 7 SMIFH2-treated mice; mean and SEM are adjacent to each scatter plot; no significant differences were detected between groups (*p* = 0.2), statistical analysis performed by two-tailed Wilcoxon test. All graphs: data presented as mean ± SEM, and source data are provided as source data file.

We next asked whether INF2-driven actinification plays a role in neuronal survival following ischemic stroke. Focal stroke was induced using single vessel photothrombosis in mouse cortex. Brains were fixed 4 h after induction of vessel occlusion, and Fluorojade C was used as a marker of early cell death in post-fixed histological sections to evaluate the effect of inhibiting formin activation using the compound SMIFH2. SMIFH2 or vehicle was incorporated into an agarose layer overlying the cortical surface during preparation of an acute cranial window ~0.5 h prior to vessel occlusion, and ~4.5 h prior to perfusion fixation. We found that pre-treatment with the formin inhibitor induced a near doubling of cell death after stroke, compared to that observed with vehicle treatment (Fig. 7b and Supplementary Fig. 10). Neither vehicle nor SMIFH2 induced detectable FluoroJade C staining in the absence of stroke.

Quantification of the ischemic volume indicated that formin inhibition induced a non-significant trend toward increased infarct volume at 4 h post-occlusion (Fig. 7c, d). Moreover, we observed a substantial increase in apparent damage to cortical tissue within the core of the infarct, with variably sized cavities appearing after strokes induced in the presence of SMIFH2. Such cavitation was never observed in non-infarcted brain regions nor in vehicle treated brains either with or without stroke. Despite these indicators of enhanced tissue damage following stroke, SMIFH2 did not induce a significant increase in the leakage of immunoglobulin from compromised blood-brain barrier following vessel occlusion (Fig. 7c, d). In addition, direct measurement of unoccluded arteriole diameters in the vicinity of the occluded vessel showed no differences between vehicle or SMIFH2 treatment (Fig. 7e), indicating that the drug did not alter local blood flow dynamics prior to stroke induction. These data indicate that INF2 normally helps protect ischemic brain tissue during the acute phase of infarct development.

## Discussion

Cellular pro-survival responses engage multiple subcellular events to enable cells to endure periods of transient stress^58^. Excitable cells like neurons and cardiomyocytes are especially vulnerable to osmotic stress because perturbed flux through various ion channels can lead to osmotic imbalance. Swelling in these cells and tissues endangers the survival of the organism. Due to the critical role of ATP-dependent ion pumps in osmoregulation, catastrophic reduction in cellular ATP throws such homeostatic mechanisms into disarray. Here we show that the neuronal actin cytoskeleton undergoes a rapid and dramatic reorganization in response to ischemia or excitotoxic levels of glutamate—conditions that trigger cytotoxic edema. This neuronal response is reversible and pro-survival.

**Fig. 8:**
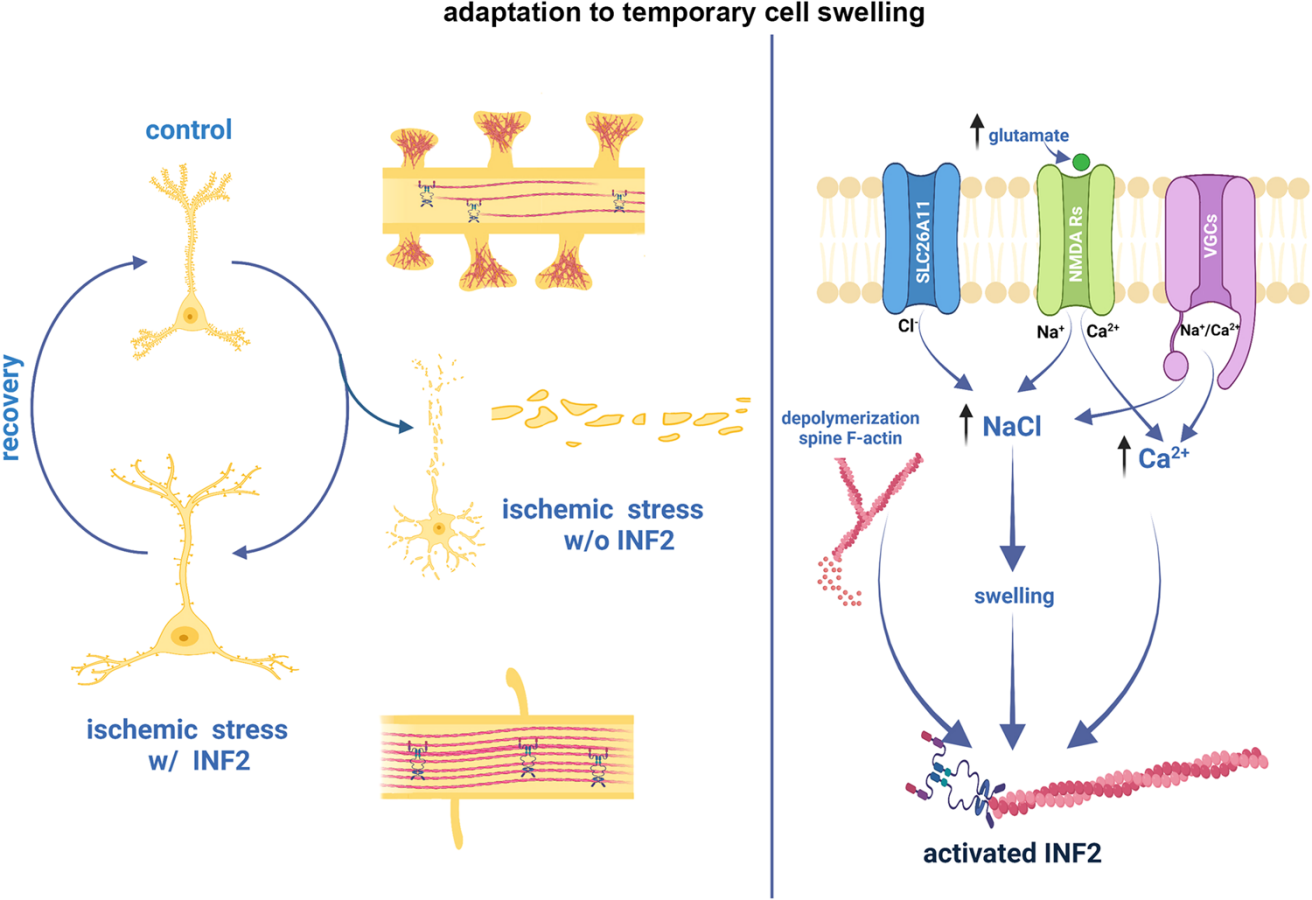
Model for the mechanism and function of ischemia-induced neuronal actinification. (Left) In response to ischemic stress, neurons undergo osmotic swelling and an extensive, but reversible, INF2-mediated reorganization of the actin cytoskeleton, characterized by F-actin disassembly in dendritic spines and F-actin accumulation in the dendrite shaft (actinification). In the absence of INF2, neurons fail to actinify and instead undergo premature cell death. (Right) Summary of key molecular steps connecting ischemia and its excess NMDA receptor activation with INF2-mediated F-actin polymerization in the soma and dendrite. Actinification is triggered by cell swelling and requires Ca^2+^ influx and depolymerization of spine F-actin. This diagram was created with Biorender.com.

Our data suggest a model (Fig. 8) in which a strong influx of sodium and chloride, and subsequent water entry (the key drivers of cell swelling), along with an influx of calcium ion, are necessary and sufficient to induce a fundamental reorganization of the actin cytoskeleton within the somatodendritic compartment of neurons. This convergence of ion influx leads to the activation of the diaphanous family formin INF2. We postulate that INF2 is required either to nucleate new filaments or to elongate existing short filaments within the soma and dendrite. The identification of a formin-based mechanism for actinification is consistent with several lines of evidence, including the long, unbranched filaments observed using both light and electron microscopy, the complete inhibition of actinification by the formin inhibitor SMIFH2, and the complete prevention of actinification by genetic silencing of INF2, which was rescued by ectopic expression of INF2. Interestingly, although we could observe actinification in live cells using multiple fluorescent reporters of actin filaments (Lifeact-mRFP, mApple-F-tractin, and the small molecule probe SiR-actin), we were unsuccessful in observing actinification using GFP-actin. This also is consistent with a formin based mechanism, since various reports indicate that large-moiety terminal tags on G-actin interfere with formin-dependent F-actin assembly^59–64^.

Importantly, several lines of evidence establish that the actinification phenomenon is distinct from the formation of cofilin-actin rods, which can also form in response to excess glutamate^55^. First, the spatial and temporal features of these events are different, with actinification occurring rapidly within 5 min and predominantly in the soma and proximal dendrites, while cofilin-actin rods reportedly appear after at least 1 h, preferentially in distal dendrites^65^, an observation that we confirmed in our own culture system. Second, we found that cofilin immunoreactivity did not colocalize with the actinified filaments as would be expected for cofilin-actin rods. Finally, multiple means of preventing or inducing cofilin activation neither prevented or favored actinification. The accumulation of F-actin within the dendrites also did not resemble typical actin stress fibers, since it was not prevented by blebbistatin. We therefore conclude that the actin filaments that accumulate within the somatodendritic compartment following osmotic stress in neurons are distinct.

The precise mechanisms by which swelling and calcium entry converge to activate INF2 require further investigation. That stress-induced activation is required is apparent from the experiments that demonstrated that overexpression of wildtype INF2 enhanced the propensity of neurons to actinify but did not by itself induce actinification, in contrast to expression of a constitutively active INF2 that by itself induced actinification even in the absence of NMDA. Higgs and colleagues have shown that, in non-neuronal cells, an inactive conformation of INF2 is maintained via binding of a complex of lysine acetylated G-actin bound to cyclase-associated protein (CAP) to the INF2 DAD and DID domains, respectively, and that INF2 becomes activated when HDAC6 activity deacetylates G-actin^54^. In our studies of primary neurons, however, none of the various deacetylase inhibitors we tested, including the HDAC6 inhibitor tubastatin, robustly inhibited NMDA-induced actinification. Nevertheless, some mechanism involving acetylation/deacetylation activity does seem to influence actinification driven by INF2, since the compound C646, which broadly inhibits acetyltransferases, by itself caused actinification, even in the presence of NMDA antagonists. This effect of C646 was blocked in the presence of the formin inhibitor SMIFH2, indicating it may act via INF2, similar to NMDA. Taken together, we cannot rule out that NMDA induces INF2 activity via a pathway involving deacetylation, but our results suggest that NMDA possibly activates INF2 through alternative mechanisms.

Studies have demonstrated in vitro and in cells that elevated actin monomer concentration can compete with INF2 autoinhibition in addition to its role as nucleation substrate^49,66^. We postulate that the abrupt rise in G-actin driven by NMDA-induced depolymerization of spine actin generates a burst of soluble monomers that may facilitate the activation of INF2. In accord with this hypothesis, prevention of F-actin disassembly by jasplakinolide completely blocked NMDA-induced actinification (Fig. 5f). If actin monomer is part of the NMDA-dependent INF2 activation mechanism, the G-actin could come from anywhere in the dendritic compartment, in principle; however, spines likely represent the largest reservoir. It is also possible that the depolymerization of F-actin (within spines or elsewhere) causes the release of a sequestered factor that either activates INF2 or promotes F-actin polymerization. Future studies are needed to probe more precisely how NMDA or ischemia induce the presumptive conformational change that relieves INF2 autoinhibition. Our studies demonstrate that calcium influx through NMDA receptors or voltage-dependent calcium channels and water entry driven by influx of sodium and chloride converge to activate INF2 either directly or indirectly.

The filaments formed during actinification appear to exhibit an unusually slow turnover. The failure of GFP-tagged forms of exogenous actin to participate in actinification precluded our ability to directly quantify actin turnover rates following NMDA. However, when latrunculin A was applied to sequester G-actin after actinification had occurred, we observed no enhanced clearance of actinified filaments for up to 2 h, suggesting that there is very little turnover of these filaments in the continued presence of NMDA. We therefore conclude that the half-time for filament turnover probably exceeds ~1 h, meaning they are highly stable. Despite this remarkable apparent stability, the filaments are able to spontaneously disassemble upon cessation of NMDA receptor activation. Although detailed characterization is required, we estimate that the half-time for filament disassembly (induced via NMDA antagonists applied after 5 min of NMDA) is on the order of 15–45 min. Interestingly, F-actin also reassembled in dendritic spines during the same time frame for F-actin disassembly in the dendrite shaft, a phenomenon that also deserves further investigation. Detailed exploration of the structure and function of both pre- and postsynaptic elements during stress and recovery, and whether any such changes are tied to actinification, will be of great interest in future studies. Here, we focused on actin filament polymerization in the dendrite and its pro-survival role.

Initially, we had assumed that the massive reorganization of F-actin represents an early step in excitotoxic neuronal cell death. However, as discussed below, we determined that actinification is pro-survival. Given that actin filament assembly is typically an energy-consuming process, it is curious that neurons would engage in large-scale F-actin polymerization during a time of cell stress, especially during hypoxia when ATP is in short supply. The ATP-dependence of actinification remains to be determined experimentally; however, polymerization of ADP-actin into filaments has indeed been described^67,68^.

The reversible nature of somatodendritic actinification suggested that it might be beneficial to the cell. Subsequent experiments convincingly demonstrated the pro-survival nature of this pathway (Fig. 6b). Prolonged incubation with NMDA induced a portion of cells to die within 1–4 h, probably via swelling and necrosis rather than apoptosis, due to its rapidity. We chose lengthy exposures to NMDA for these experiments in order to maximize cell death induction, since most neurons incubated with NMDA for shorter periods do not succumb to stress immediately, but rather over 24–48 h^69^. Note that even after 1 h of continuous exposure to NMDA only ~25–30% of control, empty vector-transfected neurons were dead at this time point (Fig. 6b, c), and only ~35% were dead after 4 h of continuous exposure to NMDA (Fig. 6b). Silencing of INF2 significantly enhanced this rapid neuronal death, and overexpression of INF2 significantly reduced it, suggesting that the level of INF2 is a key factor in the pro-survival response pathway in neurons. Intriguingly, higher endogenous levels of INF2 were predictive of a higher propensity to actinify quickly in response to NMDA (Fig. 5c), suggesting one possible mechanism underlying variable cellular susceptibility to excitotoxicity.

In addition, pharmacological inhibition of formin activity in vivo using SMIFH2 showed that blocking this pathway significantly worsened ischemic infarct severity. Caution is applied in this case because SMIFH2 can also affect some myosins^44^, and additional experiments will be required to confirm that INF2 mediates stroke severity in vivo. However, our experiments in neuronal cultures support the idea that INF2 mediates both actinification and cell survival, and blocking neither myosin II nor myosin V in culture prevents actinification, while SMIFH2 robustly does. Moreover, since reports from the literature indicate that blocking myosin II tends to be neuroprotective in stroke models^70^, it seems unlikely that these pathways are linked to our observation that SMIHF2 worsened the infarcts. Given that at early times (i.e., hours) following a stroke most of the neuronal cell death that generates the infarct is mediated by pathological cell swelling^37,71^, we postulate that INF2-mediated actinification attenuates the effects of cell swelling and reduces cell death in the early stages after a stroke.

Consistent with this hypothesis, we observed the appearance of small cavities within the core of the infarct in our experimental model of stroke, but only when formin activity was inhibited (Fig. 7). Cavitation within infarct zones has been described previously as cavities which appear many days or weeks after a stroke, evolving from a cystic, fluid filled core as cellular debris is cleared from the infarct^72,73^. The early appearance of tissue cavities was therefore unexpected, and this observation is worthy of follow up investigation. One possibility consistent with our model is that blockage of formin activity renders neurons so susceptible to edema that they undergo cytolysis, leading to rapid tissue damage and cavitation in the acute ischemic core.

The precise subcellular mechanisms by which actinification confers a pro-survival advantage remain to be determined using physiological and biophysical approaches. Given the rapidity of actinification and the fact that we can observe protection against membrane leakage (an indicator of cell death) within at least one hour of initiating incubation of cultures with NMDA, it seems unlikely that actinification and its protective actions require changes in gene expression or protein synthesis, and we indeed found that blocking translation had no effect. Instead, actinification may serve to alter the local osmotic and/or biomechanical landscape to prevent plasma membrane rupture. Indeed, given actin’s overall abundance in cells, it is possible that the extensive actin polymerization may lessen osmotic stress by reducing the concentration of free actin monomer itself, which, if not counteracted, could contribute meaningfully to total osmolyte concentration. In addition, actinification may protect organelles from osmotic stress via osmolyte regulation or local mechanical protection (i.e., forming a protective “cage” around the organelle). Probing such possibilities is a rich area for future investigation. One important question will be to determine with greater precision the temporal relationship between F-actin depolymerization from spines and the initiation of F-actin polymerization within the dendrite shaft. At our current level of spatiotemporal resolution we can only conclude that these two events occur in close temporal proximity, and we so far have been unable to determine if one begins before the other. It also remains to be determined whether actinification is initiated at specific loci within the dendrite.

The pro-survival function of INF2-mediated actinification might be selective for neuronal edema or related types of osmotic stress. We observed that actinification requires a convergence of calcium entry together with an ionic imbalance and water entry. This may imply that actinification selectively protects neurons from cytotoxic edema, but not other stressful conditions. Moreover, it will be of interest to determine whether actinification is a relevant pro-survival mechanism in widely-occurring injury conditions involving glutamate receptor overload, including traumatic brain injury and seizures in addition to hypoxia/ischemia.

Previous studies have also implicated INF2 in mechanosensitive responses of cells in culture, but the in vivo relevance of these responses has not been explored. A study in XTC cells demonstrated mechanosensitive activation and processive F-actin polymerization by diaphanous formins, including INF2^74^. A study in NIH 3T3 cells showed that INF2 mediated the formation of a perinuclear actin rim in response to mechanical stress or calcium ionophore, but did not determine the function of this actin rim structure^51^. Another study similarly reported that INF2 mediates a transient response to cell damage or strong calcium entry, with actin polymerization occurring along the ER, simultaneous with actin depolymerization at the cell periphery, a response the authors termed “calcium-mediated actin reset”^52^. Because the temporal dynamics, sodium and chloride dependence, calcium source, and other key features of the actin polymerization events described in these prior studies appear to differ from the neuronal actinification we show here, further investigations are needed to examine the relationship among these various cytoskeletal responses. Nevertheless, it is worth postulating that INF2-driven actin polymerization may function in a general pro-survival capacity to protect many types of cells from a variety of mechanical stressors, including swelling during ischemic, hypoxic, or osmotic episodes.

## Methods

### Ethical statement

All the following procedures involving animals were conducted in accordance with the US National Institutes of Health Guide for the Care and Use of Laboratory Animals and were approved by the Institutional Animal Care and Use Committee (IACUC) at UC San Diego (protocol # S07290) and Medical University of South Carolina (protocol # 3219).

### Chemicals

2-Deoxy-d-glucose (2-DG, Sigma D6134-1G); NMDA (Tocris, 0114); D-AP5 (Tocris, 0106); MK801 (Tocris, 0924); CGP (Tocris, 1493); DIDS (Sigma, D3514); Veratridine (Tocris, 2918); Bumetamide (Sigma, B3023); GlyH-101 (Sigma, 219671); NPPB (Tocris, 0593); Mannitol (Spectrum Chemical, MA165); Ionomycin (Tocris, 1704); SMIFH2 (Sigma, S4826-5MG); CK-666 (Sigma, SML_0006_5MG); Latrunculin A (Lat-A, Sigma, L5163); Thapsigargin (Tocris, 1138); Jasplakinolide (JASP, Tocris, 2792); Bovine Serum Albumin (BSA, Sigma, A4919-5G); DAPI (Biotium, 40043); SAHA (Tocris, 4652); Santacruzamate A (Tocris, 7191); CI994 (Tocris, 2952); FK228 (Tocris, 3515); Tubastatin (Tocris, 6270); Nicotinamide (Tocris, 4106); EX527 (Tocris, 2780); anti-sirtuin 5 inhibitor peptides (Dr.C.A. Olsen, NRE139; NRD167); Suramin (Fisher, AC328540500); C646 (Sigma, SML0002); Cycloheximide (Sigma, C4859); Blebbistatin (Sigma, B0560); Taxol (Tocris, 1097); Nocodazole, (Tocris, 1228); MyoVin-1, (Calbiochem, 475984); Mibefradil, (Tocris, 2198); ω-Conotoxin GVIA, (Tocris, 1085); NBQX (Tocris, 0373); Nifedipine, (Tocris, 1075). Each chemical was dissolved according to the manufacturer’s guidelines, either in water or DMSO, or ethanol.

### Primary rat neuronal culture

Rat hippocampal neurons were isolated according to ref. 75. In brief, hippocampi were dissected from brains of Sprague-Dawley rat (Charles River, 400) female and male embryos at embryonic day 19 and dissociated into individual cells by incubating in a papain-containing solution (Worthington Biochemical Co., LS003124). The cells were then washed and plated either on poly-L-lysine-coated (Sigma, P9155-5mg, 100 µg/ml) glass coverslips (Carolina Biological Supply Co., 633009) or on PEI (1:100 in borate buffer) + Laminin (Gibco, 23017015, 20 µg/ml) 24-well ibiTreat (ibidi, 82426) at a density of 500 cells/mm^2^ in Neurobasal Medium (Gibco, 21103-049) supplemented with Neurocult-SM1 neuronal supplement (Stem Cell Technology, 05731) and 0.5 mM L-glutamine (Sigma, 07100).

Neurons were usually transfected at 21 days in vitro (DIV) using Lipofectamine 2000 (Invitrogen, 11668-019). Cells were fixed or used for live cell-imaging experiments 1–2 days post-transfection, or 7–10 days post-transfection for the shRNA experiments.

### In vivo model

One-to-two-month-old C57BL/6J (The Jackson Laboratory, 000664) and Thy1-YFP-H mice (The Jackson Laboratory, 003782), both male and female, were received from Jackson laboratory, and housed on a 12 dark/12 light cycle at temperatures of 20–26 °C (set point 22.8 °C) with 30–70% humidity (set point 50%) and used for the in vivo experiments described in Fig. 1d–f, Supplementary Figs. 1–5 and in Fig. 7 and Supplementary Fig. 8, respectively. The Thy1-YFP-H mice, as previously reported, have YFP expression restricted mainly to subsets of layer V neurons facilitating the identification of dendrites and dendritic spines^76^.

### Plasmid construction for INF2 RNA interference

An shRNA previously demonstrated to knock down INF2 expression^77^ along with a control blank insert were cloned into a vector co-expressing farnesylated mApple (Addgene #54899). The shRNA and mApple were under control of the H1 and PGK promoters respectively. In-fusion cloning was used to combine the regions of the original construct and the farnesyl-mApple into an AAV backbone. The following three sets of in-fusion primers were designed flanking the m-apple farnesyl and the shRNA and blank portions of the original plasmids:

shRNA

Forward: CTATACGAAGTTATGGTCGACGGTATCGATAAGCTTG;

Reverse: GGTGGCGACCGGTAAGCTA.

m-Apple

Forward: TTACCGGTCGCCACCATGGTGAGCAAGGGC;

Reverse: TTGATTCATAACTTCTCAGGAGAGCACACACTTG.

WPRE

Forward: GAAGTTATGAATCAACCTCTGGATTAC; Reverse: GTGGCAATGCCCCAA.

### In vivo cortical microinfarct induction

The animal’s head was affixed to a stable imaging apparatus under the two-photon microscope.

A green laser light (532 nm) was used to induce single vessel photothrombosis. The laser greatly under-fills the back aperture of the 20x objective (Olympus; XLUMPlanFI), yielding a fixed laser focus with ~20–40 μm diameter at the imaging plane. The power of the green laser was ~1 mW at the sample. We administered 25 μl of 5% w/v fluorescein-dextran (Sigma, 2 MDa) into the infraorbital vein over a period of 20 s. The vasculature was clearly visible with two-photon imaging immediately after injection. The filter set used to detect fluorescein-dextran emission was 525/70m-2P (Chroma Corp). When finding a capillary location of interest for sham experiments, we targeted a region at least 20 μm away from larger penetrating vessels. After injecting 25–50 μl of 1.25% Rose Bengal (Sigma, 330000) into the infraorbital vein, using the same method described above for fluorescein-dextran we initiated photothrombosis by activating the green laser (1 mW at the sample) over a single penetrating arteriole target and allowed irradiation for 60–90 s. Vessel occlusion was assessed by direct visual verification. In sham animals, green laser irradiation of capillary locations at a similar power, led to no visible effects on the target vessel in the absence of rose Bengal.

### In vivo agarose controlled drug delivery

We generated acute, skull-removed cranial windows. Under 4% isoflurane anesthesia, we first injected 50 μl of 0.06 mg/ml buprenorphine (0.1 mg/kg for a 30 g mouse) intraperitoneally. Once the animal was in the surgical plane of anesthesia (4% MAC induction, 1–2% during surgery) the scalp was excised and periosteum cleaned from the skull surface. C&B MetaBond quick adhesive cement (Parkell; S380) was then applied to the skull surface to affix a custom-made metal flange to the right half of the skull. This metal flange could later be screwed into a custom holding post for head-fixation during imaging. A 3 mm diameter circular craniotomy (dura intact) was created over the left hemisphere, and centered over 1.5 mm posterior and 3 mm lateral to bregma, which encompasses the barrel and other regions of the somatosensory cortex. The cortical surface was cleaned of any blood and covered with a drop of warm 1.5% agarose dissolved in modified artificial cerebral spinal fluid^78^, and immediately overlaid with a 4 mm diameter glass coverslip (Warner Instruments; 64–0724 (CS-4R). Care was taken not to compress the cortex during this process, which could affect cortical microvascular flow. MetaBond was then used to seal the edges of the coverslip, and to cover any remaining exposed skull surface or skin. We opted to use topical SMIFH2 application, rather than systemic injection. This was done to avoid confounding reductions in cerebral perfusion pressure that can occur with systemic dosing. SMIFH2 was dissolved in DMSO and then added to the warm 1.5% agarose solution (0.1% DMSO concentration) used in the cranial window procedure, providing direct access to the brain through the cortical surface. The final SMIFH2 concentrations in the agarose (150 μM) was chosen based on: (1) on the concentrations we tested on dissociated neurons in culture and (2) a study showing that ~10% of similarly-sized molecules at the meninges enters the brain parenchyma through transcranial diffusion^79^. Photothrombosis was initiated 30 min after starting the SMIFH2/vehicle loaded agarose application to allow time for diffusion of the drug through the cortex. Animals were perfused transcardially with 4% paraformaldehyde in phosphate-buffered saline (PBS), pH 7.4, 4 h post-stroke to harvest the brain.

### In vivo two-photon imaging of cortical pial vessels

Cortical pial vessels were imaged from vehicle-treated and SMIFH2-treated mice. To measure the width of the arteriole diameter we used VasoMetrics a program developed in A. Y. Shih’s laboratory^80^.

### In vitro induction of actinification by bath applied NMDA

Three-week-old dissociated hippocampal cultures were incubated with either vehicle (H_2_O) or 50 µM NMDA added directly to their conditioned medium at 37 °C for 5 min or other times, as indicated. Neurons were kept in the culturing incubator prior to fixation. This approach was chosen over replacing conditioned media with fresh media containing NMDA due to the established toxicity of this manipulation^81^. The following drugs were bath applied to the cultures at the following final concentrations and incubation times: SMIFH2 70 µM 2 h, CK666 50 µM 2 h, APV 100 µM 15 min prior NMDA, MK- 801 25 µM 15 min prior NMDA, C646 25 µM (30 min), Lat-A 2 µM, DIDS 500 µM (30 min), bumetanide 100 µM (30 min), GlyH-101 50 µM (30 min), NPPB 200 µM (30 min), JASP 4 µM (5 min), taxol 10 nM (30 min) [qualitatively similar results were obtained when taxol was added at higher concentration (100 nM) and for longer (1 h)], nocodazole 10 µM (30 min), veratridine 50 µM (5 min), MyoVin 30 µM (30 min), blebbistatin 100 µM (30 min & 6 h), thapsigargin 1 µM (2 min, 30 min & 2 h), cycloheximide 35 µM (30 min), NBQX 20 µM (20 min), ω-conotoxin 10 µM (20 min), CGP 1 µM (20 min), nifedipine 30 µM (20 min), mibefradil 10 µM (20 min) and ionomycin 5 µM (30 min). For HDAC inhibitors (HDI) concentrations and incubation times, see Table 1.

### Ca^2+^ and Na^+^ depletion experiments

Conditioned medium was replaced either with calcium free solution in mM: HEPES 20, NaCl 137, MgSO_4_ 0.4, MgCl_2_ 0.5, KCl 5, KH_2_PO_4_ 0.4, NaH_2_PO_4_ 0.6, NaHCO_3_ 3, Glucose 5.6, EGTA 0.020, pH 7.3 (NaOH); or with sodium free solution: N-methyl-D- glucamine chloride 140.6, HEPES 20, MgSO_4_ 0.4, MgCl_2_ 0.5, KCl 1.4, KH_2_PO_4_ 1, KHCO_3_ 3, glucose 5.6, CaCl_2_ 1.8. pH 7.3, 320 mOSM. 50 µM NMDA plus 10 µM glycine were then added to induce actinification.

### Oxygen and glucose deprivation

To induce oxygen and glucose deprivation, 2-deoxyglucose was added to the cultures just before placing them into a hypoxic chamber XVIVO system (Biospherix, Parish, NY) at 37 °C containing 1% O_2_.

### Neuronal cell culture transfection

Lipofectamine 2000 was used to transfect 3-week-old dissociated hippocampal cultures, using the following plasmids: mApple-F-tractin (Dr. H. N. Higgs), Lifeact-mRFP (Dr. Ray Truant), Lck-GFP (Addgene plasmid 61099), mem-mApple Empty vector and mem-mApple-INF2-shRNA (see above section about plasmid construction for INF2 RNA interference), GFP-INF2-wt (CAAX & non-CAAX) (Dr. H. N. Higgs), peGFP-N1 (Clontech), GFP-empty v., GFP-INF2-shRNA, GFP-INF2-CA (Dr. H. N. Higgs).

Fresh Neurobasal Medium was used to prepare the mixture of Lipofectamine and cDNA. In all, 1 µl of Lipofectamine and 1.5 µg of cDNA per 50 µl of NBM were mixed, incubated for 30 min and then added dropwise to 500 µl NBM in which neurons had been growing for 3 weeks. Washing was not required and neurons showed no overt signs of toxicity.

### Immunostaining

Hippocampal cultures were fixed with 3.7% formaldehyde in phosphate-buffered saline (PBS) plus 120 mM sucrose for 20 min at 37 °C. Samples were incubated in 20 mM glycine for 5 min, rinsed and permeabilized with 0.2% Triton X-100 for 5 min at room temperature, and then blocked for 30 min with 2% bovine serum albumin (BSA). All primary antibodies (listed at the end of this section) were incubated for 1 h at room temperature, and, following rinsing with PBS, were incubated with AlexaFluor-conjugated secondary antibodies (Invitrogen, Molecular Probes) for 45 min at 37 °C. To label F-actin, AlexaFluor488-, 568- or 647-phalloidin at 1∶1000 (Invitrogen, Molecular Probes, A12379, A12380, A22287) was incubated for 2 hr at room temperature in the presence of 2% BSA. Finally, the coverslips were washed twice with PBS and mounted using either Aqua-Mount (Learner Laboratories, 13800) for acquisition with regular spinning disk confocal or Prolong Gold Antifade (Molecular Probes, P36934) for Airyscan acquisition.

To stain for cofilin-actin rods cultures were processed according to procedures modified from ref. 82. Cultures were fixed for 45 min, at room temperature, in 4% formaldehyde in phosphate-buffered saline (PBS) + 0.1% glutaraldehyde. Neurons were then permeabilized with −20 °C cold methanol for 3 min, and then blocked with 5% goat serum/1% bovine serum albumin in TBS (10 mM Tris pH 8.0, 150 mM NaCl). Anti-actin (1:300) and anti-cofilin (1:300) antibodies were incubated overnight at 4 °C, together with anti-MAP2 (1:500). After rinsing with TBS, neurons were incubated with AlexaFluor-conjugated secondary antibodies (1:250, Invitrogen, Molecular Probes) for 1 h at RT. Finally, the coverslips were washed again with TBS and mounted using ProLong Gold Antifade (Molecular Probes).

For brain section immunohistochemical analysis, harvested brains were placed into 30% sucrose overnight and shipped to the Halpain lab where the brains were sliced into 30 µm-thick coronal sections by using a vibratome, and stored in PBS. After blocking for 2 h at room temperature with 20% normal goat serum (NGS) and 0.3% TritonX-100 in PBS, sections were incubated with primary antibodies in 10% NGS and 0.3% TritonX-100 for 24 h at 4 °C on an orbital shaker. Afterwards the sections were rinsed in PBS three times over a period of three hours, before incubation with secondary antibodies overnight at 4 °C. Finally, the floating sections were transferred from the solution with the secondary antibodies to a solution with phalloidin and DAPI for the last 24 h before washing and mounting them with Aqua-Mount (Lerner Laboratories 13800) on positive charged slides. Alexa Fluor 647 anti-mouse IgG (1:100, Invitrogen A21237) was added with the secondary antibodies to detect the vascular leakage.

For immunostaining the following primary antibodies were used: Rabbit polyclonal anti-INF2 (1:100, Millipore Cat# ABT61 and Higgs lab DT157); Chicken polyclonal anti-Map2 (1:500, Lifespan Biosciences Inc., Cat# LS-B290); Chicken polyclonal anti-NeuN (1:200, Millipore Cat# ABN91); Mouse monoclonal anti-NeuN (1:100, Millipore Cat# MAB377); Rabbit anti-cofilin (1:300, Abcam ab11062); Mouse anti-actin (1:300, Clone C4) Millipore MAB1501; Anti-Hypoxyprobe (1:500, HP3-1000Kit Hypoxyprobe); Rat monoclonal anti-HA High Affinity, clone 3F10 (1:100, Millipore Cat# 11867423001).

For immunostaining the following secondary antibodies were used: anti-mouse IgG antibody Alexa Fluor 647, Goat (Invitrogen Cat# A21237, 1:250); anti-mouse IgG antibody Alexa Fluor 488, Goat (Invitrogen Cat# A11001, 1:250); anti-mouse IgG antibody Alexa Fluor 568, Goat (Invitrogen Cat# A11031, 1:250); anti-rabbit IgG antibody Alexa Fluor 568, Goat (Invitrogen Cat# A11011, 1:250); anti-rabbit IgG antibody Alexa Fluor 488, Goat (Invitrogen Cat# A11008, 1:250); anti-rabbit IgG antibody Alexa Fluor 647, Goat (Invitrogen Cat# A21244, 1:250).

### Platinum replica electron microscopy

Sample preparation for platinum replica electron microscopy (PREM) was performed as described previously^83,84^. In brief, detergent-extracted samples were sequentially fixed with 2% glutaraldehyde in 0.1 M Na-cacodylate buffer (pH 7.3), 0.1% tannic acid, and 0.2% uranyl acetate; critical point dried; coated with platinum and carbon; and transferred onto 50 mesh electron microscopic grids for observation. Detergent extraction was done with 1% Triton X-100 in PEM buffer (100 mM Pipes-KOH, pH 6.9, 1 mM MgCl_2_, and 1 mM EGTA) containing 2% polyethelene glycol (molecular weight of 35,000), 2 μM phalloidin, and 10 μM taxol for 3 min at room temperature. Samples were analyzed using JEM 1011 transmission electron microscope (JEOL USA, Peabody, MA) operated at 100 kV. Images were captured by ORIUS 832.10 W CCD camera (Gatan, Warrendale, PA). PREM images are presented in inverted contrast. Color labeling was performed using Hue/Saturation tool in Adobe Photoshop CC (2017.1.1 release) software to avoid obscuring the structural details. Neurons for PREM were purchased from Neurons R Us at Penn Medicine Translational Neuroscience Center (PTNC) at the University of Pennsylvania.

### Image acquisition of fixed specimen

Unless otherwise indicated, all fluorescence images shown in this article represent maximum projection images derived from a *z*-stack.

To acquire images of fixed dissociated cultures or brain sections we used an Olympus IX-70 microscope equipped with a CSU-X1 spinning disk confocal (Yokogawa Electric Corporation) custom equipped with 405 nm, 491 nm, 561 nm and 640 nm 50 mW solid state lasers (Solamere Technology Group Inc.) and a CoolSNAP HQ2 digital CCD camera (Photometrics) with pixel size of 91 nm. Fluorescence emission was selected through the following bandpass filters: 525/50 nm, 595/50, 700/75. Metamorph (Molecular Devices) was used to acquire a stack of 6–11 images in the *z*-dimension using optical slice thickness of 0.2 µm for 60X images of dissociated cultures and brain sections. A single plane of focus was used to acquire low magnification images of brain sections using a ×1.25 objective.

### Super-resolution imaging

A Zeiss LSM 880 Rear Port Laser Scanning Confocal with Airyscan FAST Microscope with a ×63/1.4 oil objective and the Zen lite software (Zeiss) was used to acquire super-resolution images of actinified neurons (Fig. 2a).

### Time-lapse imaging

Live images were acquired every 30 s before and after NMDA-induced actinification or every 15 min when recovery from actinification was monitored with image acquisition times of 0.01–0.2 s using a Nikon Ti-E microscope with perfect focus system (Nikon) and an iXon X3 DU897 EM-CCD camera (Andor Technology plc). The microscope was equipped with a CSU-X1 spinning disk confocal (Yokogawa Electric Corporation), and a customized CO_2_-delivery, temperature-controlled chamber (5% CO_2_, 35 °C). A ×601.4 NA Plan APO oil immersion objective was used for all experiments. Fluorescent specimens were excited using a laser launch (Solamere Technology Group Inc.) equipped with 488 nm, 561 nm and 640 nm 100 mW solid state lasers. Fluorescence emission was selected through the following band-pass filters: 525/50 nm, 595/50, 700/75. Metamorph (Molecular Devices) was used to acquire a stack of images in the *z* dimension using optical slice thickness of 0.2 µm; during live imaging the number of *z*-sections was typically kept to ≤4 to minimize photodamage. Only experiments in which focus was precisely maintained throughout the recording session were included in the time-lapse analyses. Experiments that required imaging neurons for long durations during cell stress, such as monitoring recovery after actinification, required neurons to be cultured and transfected on 35 mm glass bottom petri-dishes (Matsunami) for use with our Tokai HIT CO_2_-delivery, temperature-controlled chamber (5% CO_2_, 35 °C), with an embedded heated water bath to tightly control humidity and temperature. Supplemental Movies 1 and 2 were acquired on a Nikon system equipped with CSU-W1 spinning disk, photometrics prime 95B sCMOS camera and Nikon Live SR optical super resolution module.

### Cell viability assays

In dissociated cultured neurons VivaFix reagent (Biorad, 135–1118) was added directly to the conditioned media and cultured neurons for 20 min at 37 °C, modifying manufacturer’s protocol (Biorad). Samples were rinsed once with conditioned media from sister cultures before live imaging or fixing the cells, and co-staining with phalloidin or other markers where indicated.

Fluoro-Jade C (Millipore, AG325-30MG) staining was used to assess cell viability in brain sections. Free-floating brain sections were first incubated with the anti NeuN antibody for 24 h. Then they were mounted onto charged slides, which were dried for 30 min at 50 °C, then rinsed for 5 min in distilled water, incubated in 0.06% potassium permanganate solution for 5 min and rinsed again in water. Slides were then transferred for 10 min to a 0.0001% solution of Fluoro-Jade C dissolved in 0.1% acetic acid. DAPI was added at this step. The slides were then rinsed through three changes of distilled water for 1 min per change. Excess water was drained, and slides were then air dried on a slide warmer at 50 °C for at least 5 min. The air-dried slides were cleared in xylene for at least 1 min and then coverslipped with DPX non-fluorescent mounting media (Sigma, 06522-100 ml).

### Image analysis procedures

In all experiments, digital images were acquired using identical parameters and settings (e.g., laser excitation power, acquisition time, time-lapse interval, exposure time, etc.) across experimental conditions. All images displayed in this paper use identical image display settings whenever experimental groups are compared to one another. Sample sizes are provided in all figure legends. For analyses we used Fiji/ImageJ 1.53s (NIH), the open source image software^85^. Adobe Systems Inc. software Photoshop CC 2017.1.1 release was used for image display, and for analysis of fixed cultures using the binary scoring system described below.

### Quantification of actin reorganization (actinification)

For our typical assays of actinification in fixed culture we used a binary categorization whereby an unbiased observer identified a neuron as either “actinified” or “non-actinified”. “Actinified” neurons were defined as showing distinct accumulation of F-actin in the soma and proximal dendrites; “non-actinified” neurons showed distinct concentration of phalloidin signal in dendritic spines, and little accumulation in the dendrite shaft. This binary mode of designation was valid because, although different individual neurons became actinified at various times following the onset of a stimulus (e.g., NMDA), once actinification was initiated it proceeded rapidly, and the filaments that accumulated in actinified neurons were stable for long periods. This subjective, binary approach to quantification was applied in an unbiased manner, because observers assigned to collect and quantify the images were blind to the experimental manipulation via randomized encoding of the samples. Sample identification was not unveiled until after all data for a given experiment were collected and analyzed.

We sampled a minimum of 20 randomly distributed fields of view in each specimen well. Such fields of view contain, on average, about 8 neurons, for a total of ~160 neurons sample per well. We combined data from two sample wells per experiment for a total of ~320 neurons per independent treatment group. We repeated each experiment using 2–3 independent culture preparations (i.e., *n* = 2–3), thereby sampling from ~640 to 960 individual neurons for each treatment group for each experiment that is depicted here using bar graphs. For actinification assays in cells transfected with cDNAs (Figs. 5d, e and 6b, c), the number of neurons sampled was ~15–35 neurons per treatment group per replicate (sampling was necessarily lower due to the low transfection efficiency that is typical for primary neuron cultures).

In Fig. 3b, actinification was quantified within the proximal region of the dendrite outlined using Fiji/ImageJ 1.53s (NIH). Briefly, the proximal region of the shaft was cropped after subtracting background. The cropped region of the shaft was then thresholded so that it would be uniformly highlighted. The thresholded area was then binarized and any feature outside the shaft was removed using the erase tool. The plugin Macros was used to outline the binarized thresholded area to generate a ROI (region of interest), which could be saved and added to the original cropped image using ROI Manager, as shown in Fig. 3c. This allowed use of Fiji/ImageJ 1.53s (NIH) to measure integrated pixel intensity for phalloidin staining only within the generated ROI.

In Fig. 4a, dendritic actinification was quantified using live cell imaging of individual neurons labeled using the genetically encoded fluorophores mApple-F-tractin, Lifeact-mRFP, or SiR-Actin (Cytoskeleton Inc., CY-SC001), which selectively label F-actin. Using time-lapse images collected over the course of 20–30 min, a region of interest (ROI), small enough to fit between spines perpendicular to the plane of focus of the shaft, was used to quantify, over time, the F-actin signal intensity within the background-corrected dendrite shaft, which increased during actinification, as quantified over time by a line scan (1 pixel wide) manually drawn across the same dendrite labeled with the co-transfected plasma membrane marker (lck-GFP). In Fig. 2c we used the Nikon software NIS-Elements AR 5.30.03 and F-actin intensity was measured inside small ROIs placed both in the dendritic shaft and inside adjacent spines within an image. The dimensions of the ROIs were determined by the ability to remain within the individual morphing spines over time or as far as the shaft, by the presence of spines perpendicular to the plane of focus.

In brain sections actinified neurons were identified within the YFP (+) neuronal population by observers blinded to the specific experimental conditions, but trained to recognize the characteristic somatic organization of the actin cytoskeleton. A neuron was defined as “actinified” if it displayed filamentous-like phalloidin staining in the interior of the soma and proximal dendrite, and “non-actinified” if it lacked clear signs of such a pattern. The quantification was organized by regions of the cortex, based on their relative proximity to the ischemic core. The core of the infarct was defined as the region with strongly reduced staining of NeuN (<90% staining intensity relative to control); the penumbra region was defined as the zone showing robust NeuN staining intensity and radially surrounding the core within 170–250 µm.

Fluoro-Jade C-positive (+) cells in selected regions of the cortex were quantified using the Fiji/ImageJ 1.53s (NIH) “analyze particles” function to automatically identify both the overall number of nuclei (DAPI) and the number of FluoroJade C and DAPI (+) cells, with values corrected for area.

To quantify the effect of the formin inhibitor SMIFH2 vs vehicle on formation of caviti74es within the infarct, we calculated the ratio of the area of the cavities (regions lacking neuropil) to the area of ischemic damage, defined as the region with robustly depleted NeuN immunostaining (the “core” of the infarct, as described above). We also quantified the area of vascular leakage as the region staining for mouse immunoglobulin G. The area of tissue damage was measured in adjacent tissue sections and the total infarct volume, *V*_t_, was calculated by *V*_t_ = (*A*_1_ + *A*_2_ + … + *A*_*n*_) *h*, where *A*_*n*_ was the area of damage in the *n*th slice, and *h* was the distance between adjacent sections.

### Statistics and reproducibility

All results reported here were observed reproducibly in at least two to three independent culture preparations; similarly, stroke experiments in vivo were repeated across multiple days using mice from multiple litters. Prior to quantitative analysis, sample identity was either encoded for blinding of the experimental group prior to analysis, or image acquisition and analysis were conducted by different people to avoid observer bias. Statistical significance was set at the 95% confidence level (two tailed) and calculated using Prism 7.02 (Graphpad Software Inc.). Values are presented as the mean ± SEM.

Pharmacological and genetic experiments were statistically compared to their corresponding vehicle or wild type control constructs, which were included in each experimental replicate. Unless otherwise stated in the figure legends, bar graphs are used to display the mean and standard error of the results, and dots are overlaid onto the bars to depict the value obtained in each independent experimental replicate, which, as described above, was derived from sampling dozens (transfected cells) to hundreds (non-transfected cells) of individual neurons in each separate repetition. Statistically, because we are comparing a small number of replicates, we used mainly non-parametric ANOVAs (i.e., Kruskal–Wallis) followed by appropriate post hoc tests for multiple comparisons) and Mann–Whitney test. Where required we used two-way ANOVA, also with appropriate post-hoc tests and corrections for multiple comparisons. We report in each figure legend the tests selected and the p values obtained (and F values for two-way ANOVAs).

Representative images of non quantitative experiments are from the following number of independent experiments/biologically independent animals: Fig. 2d*n* = 12 live recordings; Fig. 2e*n* = 1; Fig. 2a*n* = 3; Fig. 3c*n* = 2; Fig. 3g*n* = 3; Fig. 5b*n* = 3; Fig. 6a*n* = 3; Fig. 6d*n* = 3; Fig. 7a*n* = 10; Fig. 7c*n* = 7 (veh + stroke); 8 (SMIFH2 + stroke); Supplementary Fig. 1*n* = 3; Supplementary Fig. 2a*n* = 41; 2b *n* = 6; Supplementary Fig. 3*n* = 3; Supplementary Fig. 4*n* = 3; Supplementary Fig. 5*n* = 3 (sham); *n* = 2 (2 h post-stroke) *n* = 3 (6 h post-stroke); Supplementary Fig. 7*n* = 2; Supplementary Fig. 9a*n* = 2; 9d *n* = 3; Supplementary Fig. 10*n* = 7.

### Reporting summary

Further information on research design is available in the Nature Research Reporting Summary linked to this article.

## Supplementary information

Supplementary Information

Description of Additional Supplementary Files

Supplementary Movie 1

Supplementary Movie 2

Reporting Summary

## Data Availability

All data supporting the study are available within the article and its Supplementary Information files. Source data are provided with this paper.

## Source data

Source Data
