# Supplementary material for: INF2-mediated actin filament reorganization confers intrinsic resilience to neuronal ischemic injury
Source: Nat Commun. 2022 Oct 13;13:6037. doi: 10.1038/s41467-022-33268-y (PMC9558009; doi:10.1038/s41467-022-33268-y)
Supplement: Supplementary file 1 — Supplementary Information [file 41467_2022_33268_MOESM1_ESM.pdf]

Supplementary information

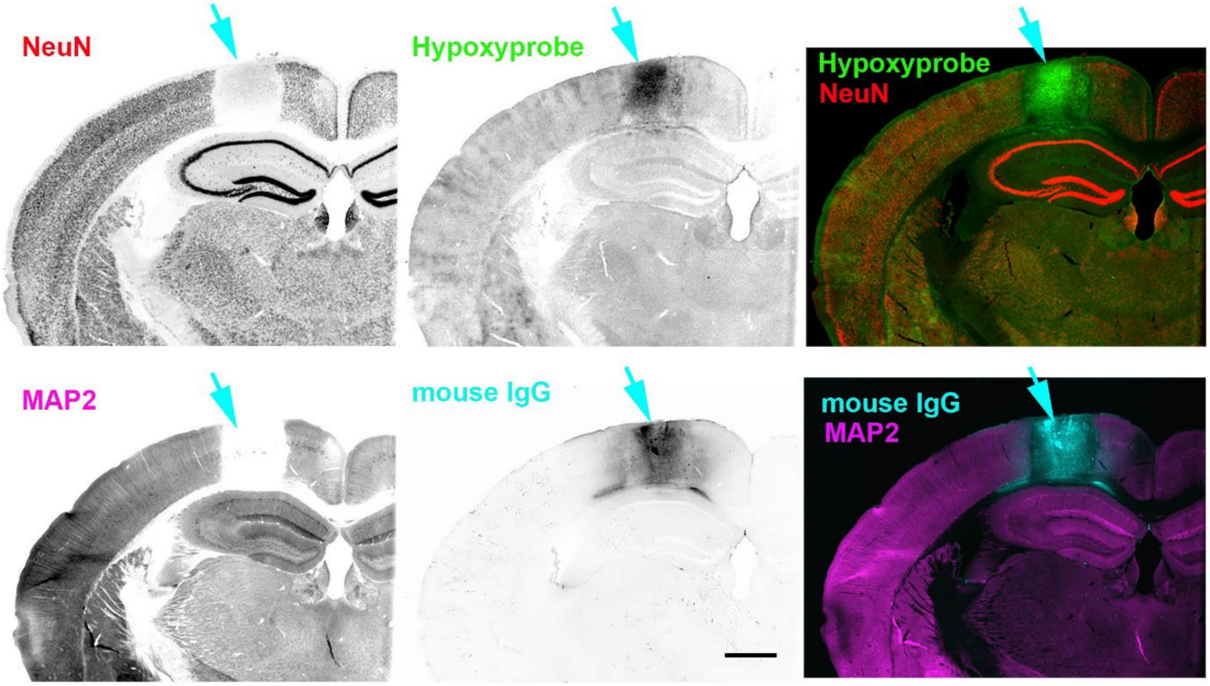

Supplementary Fig. 1 Selected histological markers of ischemic brain injury. Representative images of a single individual section of a mouse brain subject to single vessel photothrombotic stroke, immunostained with some of the indicated markers used in this study to characterize ischemic changes: anti-NeuN (specifically identifies neuronal cell bodies); anti-MAP2 (specifically identifies neuronal dendrites); hypoxyprobe (which uses an antibody to detect the presence of pimonidazole, which accumulates in tissues where pO_2 < 10mmHG); and mouse anti-IgG (to assess the extent of hypoxia-induced vascular leakage and/or blood-brain-barrier disruption). Scale bar, 700 µm.

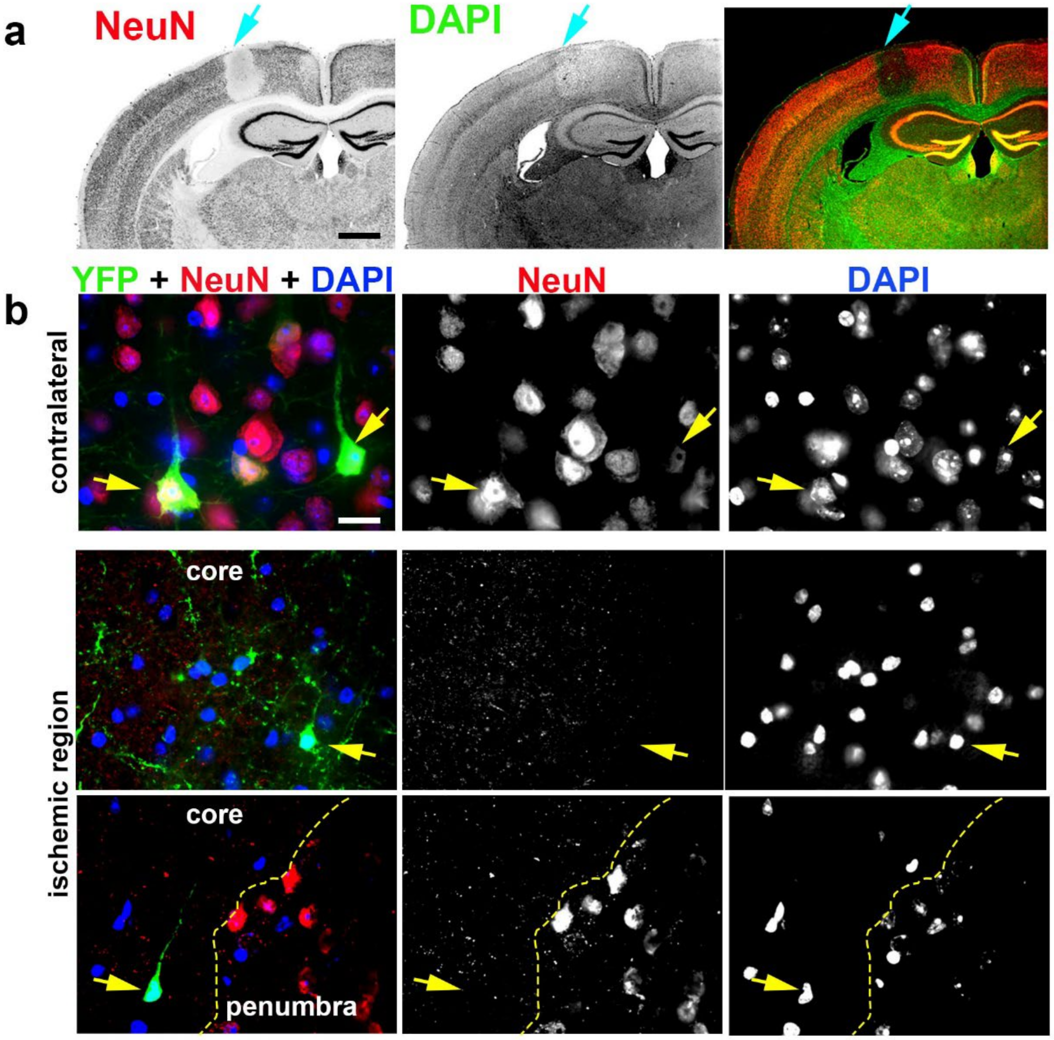

Supplementary Fig. 2 Reduction of NeuN immunofluorescence in the ischemic region does not reflect post-stroke neuronal loss. **a** Representative Thy1-YFP mouse brain section stained for NeuN and DAPI, 6h post-stroke, displaying a clear loss of NeuN signal in the ischemic core (indicated by the blue arrow), but relatively little reduction in DAPI staining. Scale bar, 1000 µm. **b** Selected regions of cortical layer 5/6 of somatosensory cortex (from the region indicated by the blue arrow in **a**) comparing at high magnification brain tissue ipsilateral vs. contralateral to the stroke. In the control, contralateral region (top row) DAPI and NeuN signals are strong and present together in all neuronal cell bodies. Arrows indicate YFP-positive neurons. In contrast, within the ischemic core (middle row), NeuN staining is lost from individual neurons while DAPI staining persists. Thy1-driven soluble YFP is detectable in neurons both within and outside the ischemic core. Dashed yellow line in the bottom row indicates the boundary where NeuN staining is lost in the ischemic core, relative to staining in the surrounding presumptive penumbra, which can be seen at this magnification as a depletion of NeuN immunoreactivity in individual neuronal cell bodies within the core, but not the penumbra. Scale bar, 30 µm.

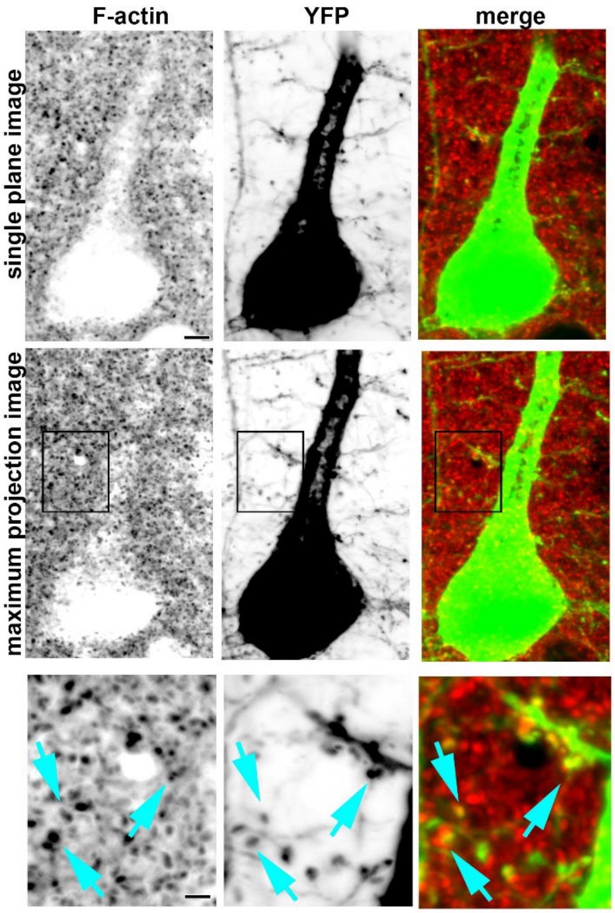

Supplementary Fig. 3 Enrichment of F-actin within dendritic spines in cortex of control mouse brain. A Thy1-YFP mouse brain was processed and stained for F-actin as described, using Alexa Fluor 647-phalloidin. Here we focus on a single YFP-positive pyramidal neuron and surrounding neuropil from Layer 5/6 in somatosensory cortex, imaged by confocal microscopy in coronal brain sections using a 60x (N.A. 1.42) oil immersion objective. Images in the left column show phalloidin staining in reverse grayscale; images in the center column show the YFP fluorescence in reverse grayscale; the right column shows a colorized merged image of the two. The top row of images shows a single plane from the z-stack of collected images. Note that dense, punctate phalloidin staining is present throughout the neuropil, but the neuronal cell body and proximal apical dendrite are relatively devoid of phalloidin staining. The middle row of images shows the same x-y field of view but as a maximum projection image from the z-stack. Note the marked increase in the number and density of phalloidin puncta, and the fact that many such puncta now overlay portions of the soma and apical dendrite. We interpret this pattern to indicate that phalloidin-positive puncta densely surround the dendrite in three dimensions, but that the interior of the somatodendritic compartment itself is relatively non-enriched for F-actin under control conditions. (We elsewhere show that stroke substantially alters this pattern, as F-actin accumulates within the somatodendrite interior; see Figure 1). The bottom row shows the boxed region at higher magnification, illustrating our consistent observation that all dendritic spines colocalize with phalloidin-positive puncta; cyan arrows point to specific examples. Given the high density of dendritic spines within mammalian cortical neuropil, we conclude that the majority of phalloidin puncta in the size range of ~0.2-1 µm in diameter that we observe are likely to correspond to dendritic spines, where F-actin is enriched. Scale bar, 4 µm upper & middle rows; 2 µm, bottom row.

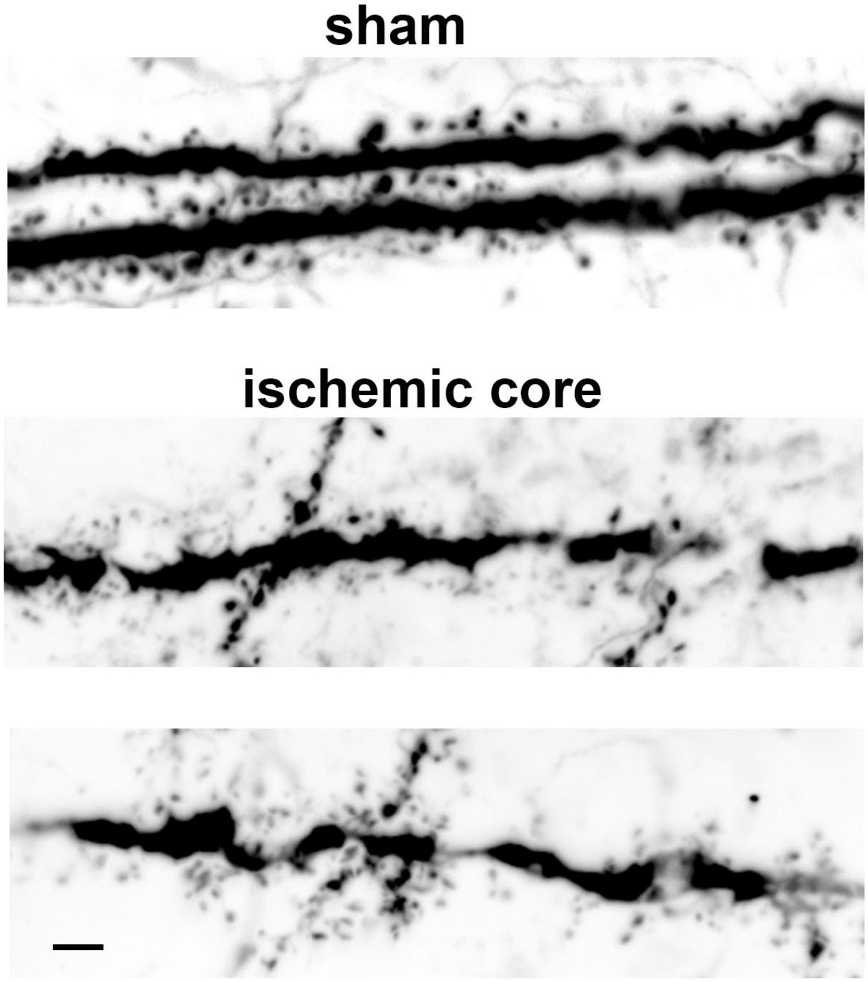

Supplementary Fig. 4 Dendritic spines decrease in size and number after stroke. Selected dendritic regions proximal to the cell bodies of Thy1-YFP-positive layer 2/3 pyramidal neurons from sham-operated vs. ischemic mouse brain. YFP fluorescence is displayed in reverse grayscale. Scale bar, 5 µm.

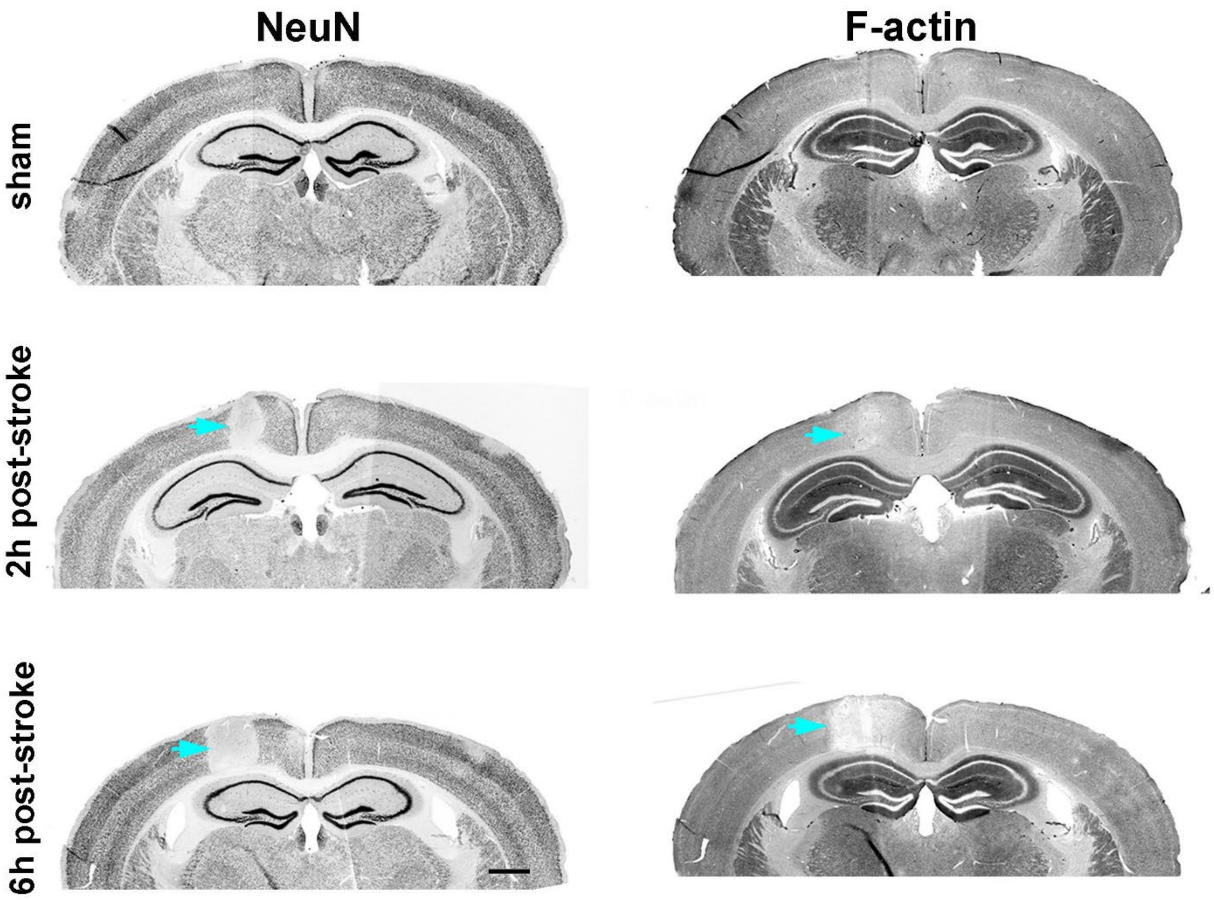

Supplementary Fig. 5. Overall F-actin signal within the ischemic core decreases over time following stroke. Representative tissue sections from sham or ischemic Thy1-YFP brains fixed at 2h and 6h after single vessel photothrombotic stroke and co-stained for NeuN and F-actin. Cyan arrows indicate the ischemic region. Note that a dramatic decrease in NeuN immunoreactivity is already observed by 2h after stroke, accompanied by a very modest decrease in phalloidin staining. By 6h post-stroke we observe that, as expected, the average infarct volume (as measured using NeuN or other markers) increases, and it is accompanied by a consistent decrease in overall phalloidin staining within the infarct zone. Nevertheless, within the infarct zone (core and penumbra) we observe numerous neuronal cell bodies with accumulations of somatodendritic F-actin that are suggestive of actinification (see Fig. 1). Scale bar, 700 µm.

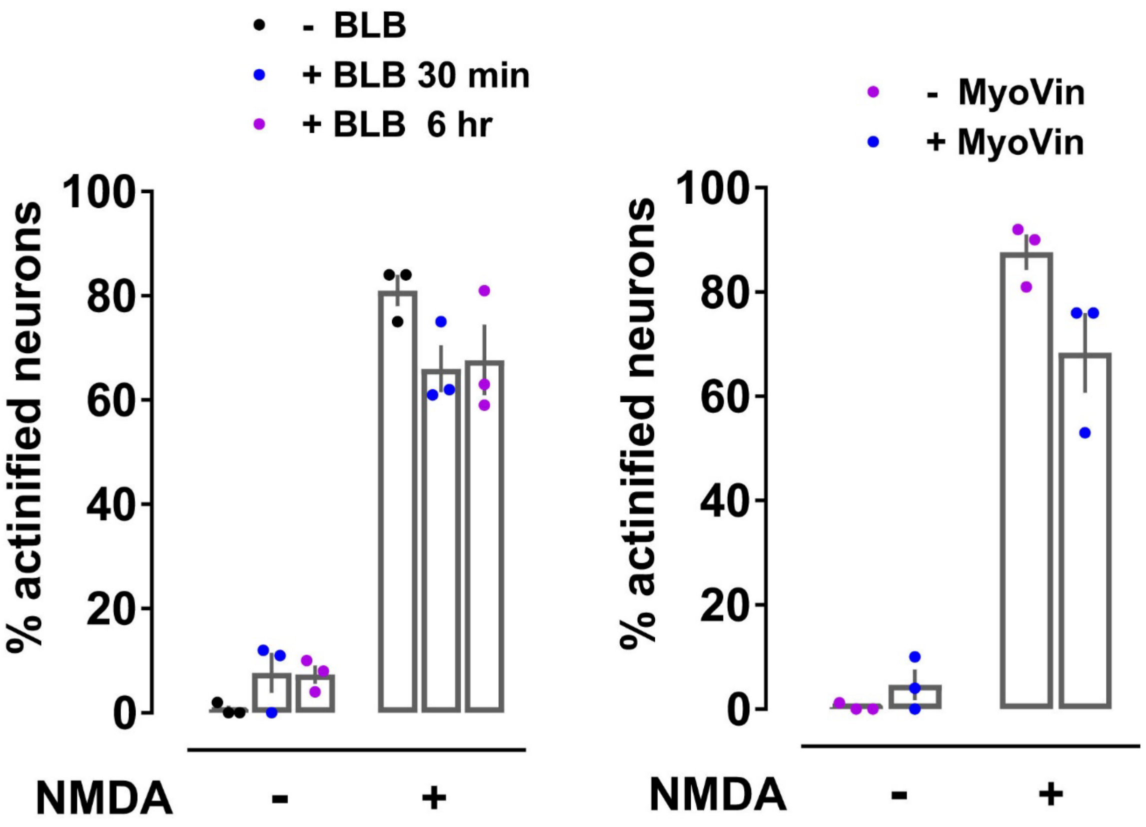

Supplementary Fig. 6 Actinification is not mediated by Myosins II and V. (Left) Quantification of the fraction of NMDA-induced actinified neurons in the presence and absence of blebbistatin (BLB), a classical myosin II inhibitor. Neurons were preincubated with 100 µM BLB for 30 min or 6 hr prior to 50 µM NMDA. Data are represented as mean ± SEM; n= 3 independent experiments; two-way ANOVA [(-)NMDA vs (+) NMDA, F (1, 12) = 423.8; p=0.0001; +/-BLB, F (2, 12) = 0.5898, n.s. p=0.5698], with Tukey's post hoc multiple comparison analysis (p<0.0001 no BLB +/- NMDA; p<0.0001 BLB 30 min +/- NMDA; p<0.0001 BLB 6h +/- NMDA; n.s. p=0.1490 NMDA +/- BLB 30 min; n.s. p=0.2337, NMDA +/- BLB 6h). (Right) Quantification of the fraction of NMDA-induced actinified neurons in the presence and absence of MyoVin, a myosin V inhibitor (30 µM, 30 min). Data are represented as mean ± SEM; n= 3 independent experiments; two-way ANOVA [(-)NMDA vs (+) NMDA, F (1, 8) = 289; p<0.0001; +/-MyoVin F (1, 8) = 2.88, n.s. p=0.1281]. Source data are provided as source data file.

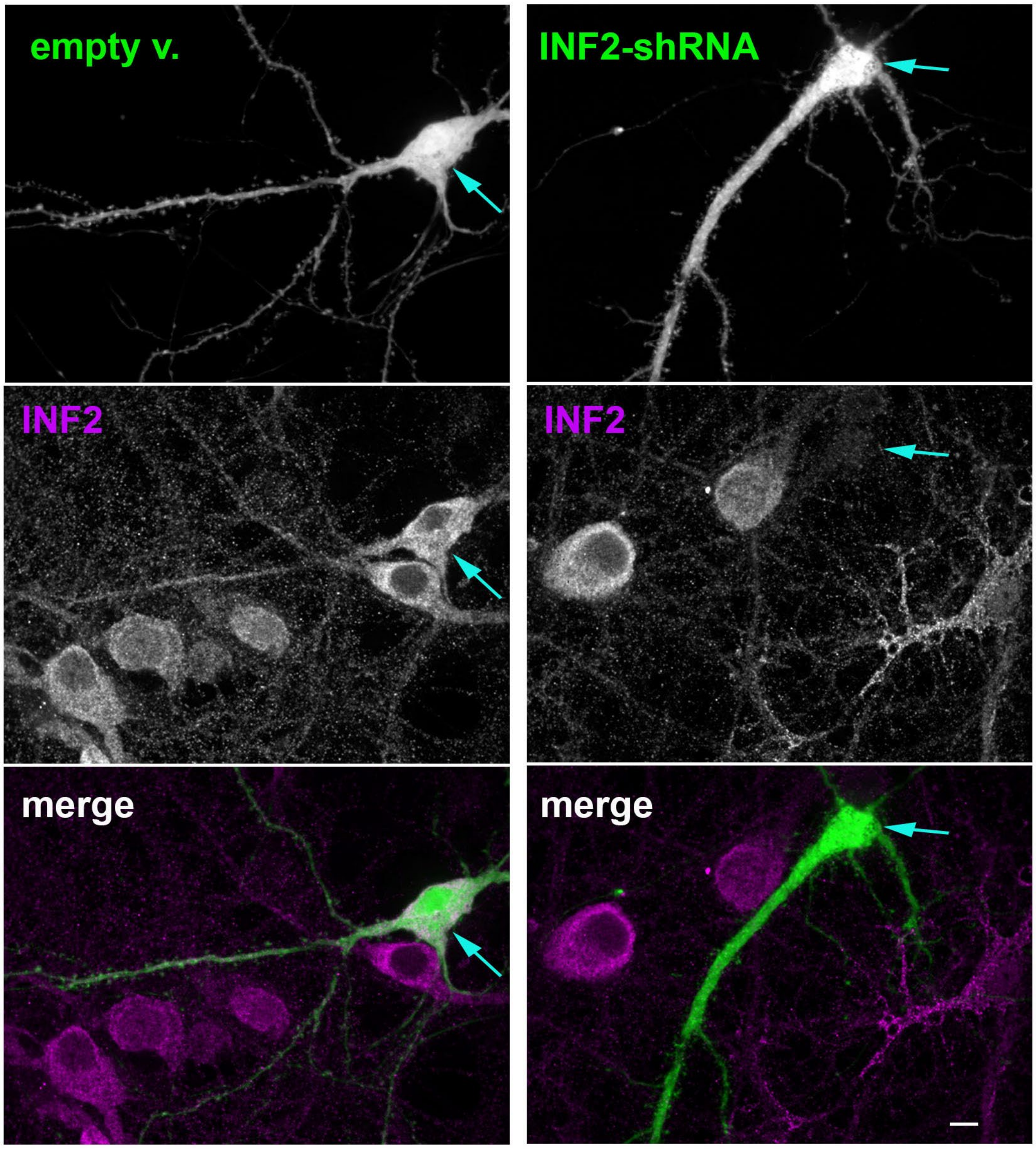

Supplementary Fig. 7 Specificity of INF2 immunoreactivity determined by RNA interference. Selected cultured hippocampal neurons transfected at DIV14 with either the empty vector (left) or the INF2-shRNA (right) for 10 days prior to fixation and immunostaining against endogenous INF2. Blue arrows point to the cell bodies of the transfected neurons and to the presence or lack of INF2 immunoreactivity in the two different experimental conditions. Scale bar, 8 µm.

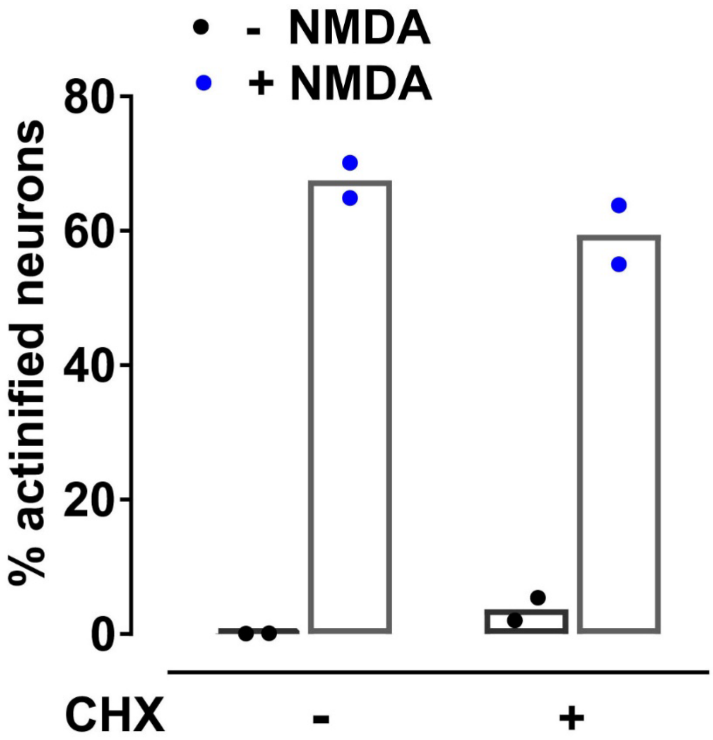

Supplementary Fig. 8 Blocking protein synthesis with cycloheximide does not prevent actinification. Neuronal cultures were incubated in the absence or presence of NMDA after a prior 30 min preincubation in the absence or presence of 35 µM cycloheximide (CHX). Data are represented as mean ± SEM; n= 2 independent experiments; two-way ANOVA [(-) NMDA vs (+) NMDA, F (1, 4) = 522.6; p=0.0001; +/- Cycloheximide F (1, 4) = 0.6903, n.s. p=0.4528], with Tukey's post hoc multiple comparison analysis (p=0.0002 no CHX +/- NMDA; p=0.0004 CHX +/- NMDA). Source data are provided as source data file.

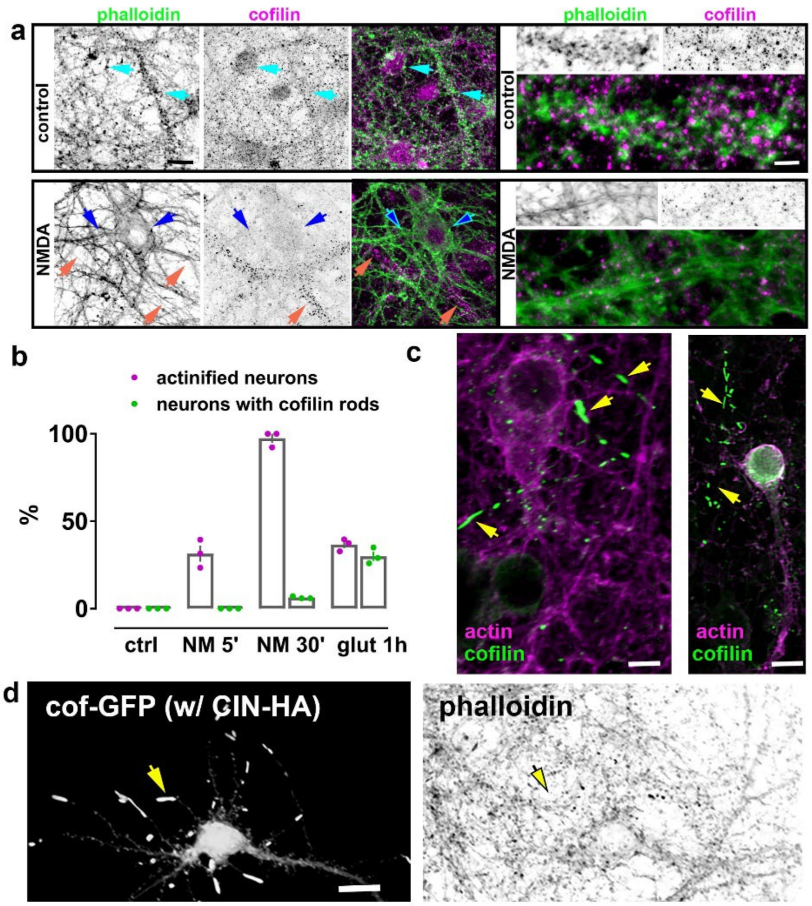

Supplementary Fig. 9 Somatodendritic actinification is a novel process distinct from cofilin-actin rod formation. **a** (Left), representative black and white images of control and NMDA-treated neurons co-stained for F-actin, and cofilin. Endogenous cofilin shows the expected punctate distribution in control neurons (cyan arrows), and little detectable immunoreactivity in NMDA-treated actinified neurons (blue arrows), while remaining detectable and punctate in neighboring astrocytes (orange arrows). Scale bar, 20 µm. (Right), selected dendritic regions from a control neuron and an actinified NMDA-treated neuron. Scale bar, 4 µm. **b** Cofilin rod formation does not occur in parallel with actinification. Cofilin rods were not detected after incubation for 5 min with NMDA, and only rarely after 30 min of NMDA, even in the actinified neurons; in contrast, we reliably detect cofilin rods after incubation with 200 µM glutamate for 1 hr or longer. Data are mean ± SEM, n=3 independent experiments; two-way ANOVA [+/-NMDA (5 & 30 min)and glut 1h, $F(3, 16) = 203.8$; $p=0.0001$; actinfied neurons vs neurons w/cofilin rods, $F(1, 16) = 425.6$, $p=0.0001$], with Tukey's post hoc multiple comparison analysis ($p<0.0001$ 5min NMDA actinified n. vs n. with cofilin rods; $p=0.0001$ 30min NMDA actinified n. vs n. with cofilin rods; n.s. $p=4349$ glut1h actinified n. vs n. with cofilin rods. Source data are provided as source data file. **c** Two representative images demonstrating the lack of colocalization between the actinified somatodendritic regions (endogenous actin stain detected using an anti-actin antibody (magenta); endogenous cofilin rods, detected using an anti-cofilin antibody (green). Scale bar, 10 µm (left image); 18 µm (right image). **d** Ectopic co-expression of cofilin and chronophin, one of its Ser-3 phosphatases, induces cofilin rods without inducing actinification. Representative neuron transfected with cofilin-GFP (Cof) and chronophin-HA (CIN) displaying the distribution of cofilin rods in distal regions of thin secondary dendrites (arrows). Scale bar, 24 µm.

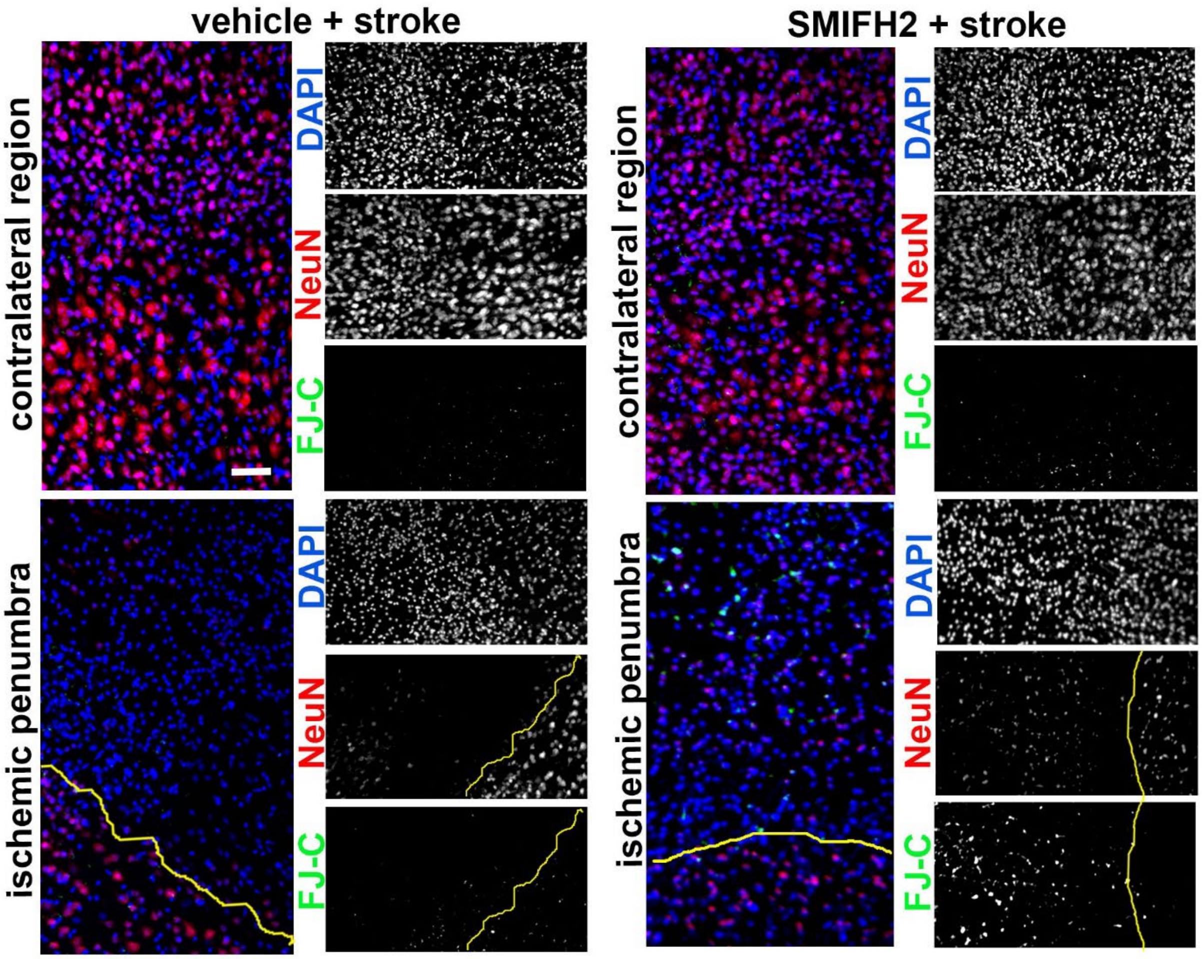

Supplementary Fig. 10 Formin inhibition increases early cell death in the ischemic penumbra. Representative cortical regions from the hemisphere contralateral to the infarct (upper half), and along the border (yellow line) between the infarct core and the penumbra (lower half), in the absence (left) or presence (right) of formin inhibitor SMIFH2 delivered by passive diffusion from a saturated agar plug placed over the thinned skull. Each color-combined image is juxtaposed to the corresponding individual grayscale channels (which are rotated 90 deg relative to the merged image). Nuclei are labeled with DAPI (in blue), NeuN (in red), and the cell death marker FluoroJade-C (FJ-C; green). Scale bar, 60 µm.
