# Supplementary material for: INF2-mediated actin filament reorganization confers intrinsic resilience to neuronal ischemic injury
Source: Nat Commun. 2022 Oct 13;13:6037. doi: 10.1038/s41467-022-33268-y (PMC9558009; doi:10.1038/s41467-022-33268-y)
Supplement: Supplementary file 2 — Description of Additional Supplementary Files [file 41467_2022_33268_MOESM2_ESM.pdf]

Supplementary Movie 1: Hippocampal neuron maintained in culture for three weeks and transfected with Lifeact-mRFP one day prior to the addition of NMDA. Acquisition was performed using a Nikon system equipped with CSUW1 spinning disk, photometrics prime 95B sCMOS camera and the Nikon Live SR optical super resolution module and a 60X water objective. The interval between frames is 30 sec; timing of NMDA addition is indicated; time stamp = min:sec. Images have been denoised and deconvolved using the Nikon NIS Elements software.

Supplementary Movie 2: Hippocampal neurons maintained in culture for three weeks and labeled with SiR-Actin prior to the addition of NMDA. Acquisition was performed using a Nikon system equipped with CSU-W1 spinning disk, photometrics prime 95B sCMOS camera and the Nikon Live SR optical super resolution module and a water 60X objective. The interval between frames is 30 sec; timing of NMDA addition is indicated; time stamp = min:sec. Images have been denoised and deconvolved using the Nikon NIS Elements software.
