# Supplementary material for: INF2-mediated actin filament reorganization confers intrinsic resilience to neuronal ischemic injury
Source: Nat Commun. 2022 Oct 13;13:6037. doi: 10.1038/s41467-022-33268-y (PMC9558009; doi:10.1038/s41467-022-33268-y)
Supplement: Supplementary file 5 — Reporting Summary [file 41467_2022_33268_MOESM5_ESM.pdf]

nature portfolio

Corresponding author(s): SHELLEY HALPAIN

Last updated by author(s): Aug 16, 2022

Reporting Summary

Nature Portfolio wishes to improve the reproducibility of the work that we publish. This form provides structure for consistency and transparency in reporting. For further information on Nature Portfolio policies, see our Editorial Policies and the Editorial Policy Checklist.

Statistics

For all statistical analyses, confirm that the following items are present in the figure legend, table legend, main text, or Methods section.

n/a	Confirmed	
☐	☒	The exact sample size (*n*) for each experimental group/condition, given as a discrete number and unit of measurement
☐	☒	A statement on whether measurements were taken from distinct samples or whether the same sample was measured repeatedly
☐	☒	The statistical test(s) used AND whether they are one- or two-sided<br>*Only common tests should be described solely by name; describe more complex techniques in the Methods section.*
☒	☐	A description of all covariates tested
☐	☒	A description of any assumptions or corrections, such as tests of normality and adjustment for multiple comparisons
☐	☒	A full description of the statistical parameters including central tendency (e.g. means) or other basic estimates (e.g. regression coefficient) AND variation (e.g. standard deviation) or associated estimates of uncertainty (e.g. confidence intervals)
☐	☒	For null hypothesis testing, the test statistic (e.g. *F*, *t*, *r*) with confidence intervals, effect sizes, degrees of freedom and *P* value noted<br>*Give P values as exact values whenever suitable.*
☒	☐	For Bayesian analysis, information on the choice of priors and Markov chain Monte Carlo settings
☒	☐	For hierarchical and complex designs, identification of the appropriate level for tests and full reporting of outcomes
☒	☐	Estimates of effect sizes (e.g. Cohen's *d*, Pearson's *r*), indicating how they were calculated

Our web collection on statistics for biologists contains articles on many of the points above.

Software and code

Policy information about availability of computer code

Data collection	Metamorph 7.10.1.161 software (Molecular Devices) was used for the acquisition of all confocal images except for experiment 2c; NIS-Elements AR 5.30.03 software (Nikon) was used to acquire the experiment in Fig.2c and all the movies; Zen lite software (Zeiss) was used to acquire the super resolution images in Fig.2a.
Data analysis	Adobe Photoshop CC 2017.1.1 release software (Adobe Creative Cloud) for color labeling of PRM images and images in Fig. 1a, b and 2a, for figure making and for analysis of fixed cultures using the binary scoring system described in Methods; NIS Elements AR 5.30.03 software (Nikon) for analysis of the experiment in Fig.2c; Fiji/ImageJ 1.53s (NIH) for the analysis of all the other experiments (see methods for details on the use of specific functions and macros); VasoMetrics (McDowell t al 2021), for measuring the width of the arteriole diameter in Fig. 7e; GraphPad Prism 7.02 (Graphpad Software Inc.) for statistical analysis.

For manuscripts utilizing custom algorithms or software that are central to the research but not yet described in published literature, software must be made available to editors and reviewers. We strongly encourage code deposition in a community repository (e.g. GitHub). See the Nature Portfolio guidelines for submitting code & software for further information.

Data

Policy information about availability of data

All manuscripts must include a data availability statement. This statement should provide the following information, where applicable:

- Accession codes, unique identifiers, or web links for publicly available datasets
- A description of any restrictions on data availability
- For clinical datasets or third party data, please ensure that the statement adheres to our policy

All the data supporting the study are available within the article and its Supplementary Information files. Source data are provided with this paper.

Human research participants

Policy information about studies involving human research participants and Sex and Gender in Research.

Reporting on sex and gender	*Use the terms sex (biological attribute) and gender (shaped by social and cultural circumstances) carefully in order to avoid confusing both terms. Indicate if findings apply to only one sex or gender; describe whether sex and gender were considered in study design whether sex and/or gender was determined based on self-reporting or assigned and methods used. Provide in the source data disaggregated sex and gender data where this information has been collected, and consent has been obtained for sharing of individual-level data; provide overall numbers in this Reporting Summary. Please state if this information has not been collected. Report sex- and gender-based analyses where performed, justify reasons for lack of sex- and gender-based analysis.*
Population characteristics	*Describe the covariate-relevant population characteristics of the human research participants (e.g. age, genotypic information, past and current diagnosis and treatment categories). If you filled out the behavioural & social sciences study design questions and have nothing to add here, write "See above."*
Recruitment	*Describe how participants were recruited. Outline any potential self-selection bias or other biases that may be present and how these are likely to impact results.*
Ethics oversight	*Identify the organization(s) that approved the study protocol.*

Note that full information on the approval of the study protocol must also be provided in the manuscript.

Field-specific reporting

Please select the one below that is the best fit for your research. If you are not sure, read the appropriate sections before making your selection.

☒ Life sciences ☐ Behavioural & social sciences ☐ Ecological, evolutionary & environmental sciences

For a reference copy of the document with all sections, see nature.com/documents/nr-reporting-summary-flat.pdf

Life sciences study design

All studies must disclose on these points even when the disclosure is negative.

Sample size	No statistical method was used to predetermine sample size. We choose the sample size in this work according to our previous studies [Calabrese et al., Neuron (PMID: 16202710); Calabrese et al., MCN (PMID: 17368908); Calabrese et al., Neuroreport (PMID: 25304495)]. The sample size as well as the number of biological repeats are provided in the relevant figure legends and methods.
Data exclusions	No data were excluded in the study
Replication	All results reported here were observed reproducibly in at least two to three independent culture preparations; similarly, stroke experiments in vivo were repeated across multiple days using mice from multiple litters, and using two different strains of mice. Note that NMDA-induced "actinification" as observed by the Halpain lab was reproduced independently by the Svitkina lab to generate electron microscopy data.
Randomization	Multiwells with primary neurons were randomly assigned to each experiment for each independent culture preparation. Observers assigned to collect and quantify the images were blind to the experimental manipulation via randomized encoding of the samples. Sample identification was not unveiled until after all data for a given experiment were collected and analyzed.
Blinding	Investigators were blinded to group allocation and sample identity both during acquisition and quantitative analysis.

Reporting for specific materials, systems and methods

We require information from authors about some types of materials, experimental systems and methods used in many studies. Here, indicate whether each material, system or method listed is relevant to your study. If you are not sure if a list item applies to your research, read the appropriate section before selecting a response.

Materials & experimental systems

n/a	Involved in the study
☐	☒ Antibodies
☒	☐ Eukaryotic cell lines
☒	☐ Palaeontology and archaeology
☐	☒ Animals and other organisms
☒	☐ Clinical data
☒	☐ Dual use research of concern

Methods

n/a	Involved in the study
☒	☐ ChIP-seq
☒	☐ Flow cytometry
☒	☐ MRI-based neuroimaging

Antibodies

Antibodies used

Anti-INF2 antibody, Rabbit polyclonal (Millipore Cat# ABT61, 1: 100, IF, RRID: AB_11203139)
Anti-INF2 antibody, Rabbit polyclonal (Higgs' Laboratory Cat# DT157, 1:100, IF)
Anti-Map2 antibody, Chicken polyclonal (Lifespan Biosciences Inc. Cat# LS-B290, 1:500, IF, RRID: AB_2138192)
Anti-NeuN antibody, Chicken polyclonal (Millipore Cat# ABN91, 1:200, IF, RRID: AB_11205760)
Anti-NeuN antibody, Mouse monoclonal, clone A60 (Millipore Cat# MAB377, 1:100, IF, RRID: AB_2298772)
Anti-cofilin antibody, Rabbit polyclonal (Abcam Cat# ab11062, 1:300, IF, RRID: AB_297714)
Anti-actin antibody, Mouse monoclonal, clone C4 (Millipore Cat# MAB1501, 1:300, IF, RRID: AB_2223041)
Anti-HA antibody, Rat monoclonal, clone 3F10 (Millipore Cat# 11867423001, 1:100, IF, RRID: AB_390918)
Anti-Hypoxyprobe antibody, Rabbit antisera (Hypoxyprobe Cat# HP3-1000Kit , 1:500, IF)
Anti-mouse IgG antibody Alexa Fluor 647, Goat (Invitrogen Cat# A21237, 1:250, IF, RRID: AB_1500743)
Anti-mouse IgG antibody Alexa Fluor 488, Goat (Invitrogen Cat# A11001, 1:250, IF, RRID: AB_2534069)
Anti-mouse IgG antibody Alexa Fluor 568, Goat (Invitrogen Cat# A11031, 1:250, IF, RRID: AB_144696)
Anti-rabbit IgG antibody Alexa Fluor 568, Goat (Invitrogen Cat# A11011, 1:250, IF, RRID: AB_143157)
Anti-rabbit IgG antibody Alexa Fluor 488, Goat (Invitrogen Cat# A11008, 1:250, IF, RRID: AB_143165)
Anti-rabbit IgG antibody Alexa Fluor 647, Goat (Invitrogen Cat# A21244, 1:250, IF, RRID: AB_2535812)

Validation

Antibodies used in this study have been validated by official website user guidance and other researchers. The detailed information can be found on the website from manufactures as listed below:

Anti-INF2 antibody, Rabbit polyclonal (Millipore Cat# ABT61). Validated by supplier using WB. https://www.emdmillipore.com/US/en/product/Anti-INF2-Antibody,MM_NF-ABT61?ReferrerURL=https%3A%2F%2Fwww.google.com%2F
Anti-INF2 antibody, Rabbit polyclonal (Higgs' Laboratory Cat# DT157). This Ab was previously validated and used for publications by the Higgs' lab. We have also validated it in this study by immunostaining after shRNA-mediated INF2 gene silencing.
Anti-Map2 antibody, Chicken polyclonal (Lifespan Biosciences Inc. Cat# LS-B290). Supplier validated this Ab for immunohistochemistry on a panel of 21 formalin-fixed, paraffin-embedded (FFPE) samples after heat induced antigen retrieval in pH 6.0 citrate buffer. https://www.lsbio.com/pathplus-antibodies/pathplus-map2-antibody-c-terminus-ihc-ls-b290/31249. We validated it by using it together with other anti-MAP2 antibodies previously published with by the Halpain lab.
Anti-NeuN antibody, Chicken polyclonal (Millipore Cat# ABN91). Validated by the supplier using WB. https://www.emdmillipore.com/US/en/product/Anti-NeuN-Antibody,MM_NF-ABN91 Also we confirm that the subcellular localization of the signal of this Ab matches other anti-NeuN antibodies and it is absent in cultured astrocytes.
Anti-NeuN antibody, Mouse monoclonal, clone A60 (Millipore Cat# MAB377). For validation supplier used brain tissue as positive control and fibroblasts (non neuronal tissue) as negative control. https://www.emdmillipore.com/US/en/product/Anti-NeuN-Antibody-clone-A60,MM_NF-MAB377 . We confirm that the subcellular localization of the signal of this Ab matches other anti-NeuN antibodies and it is absent in cultured astrocytes.
Anti-cofilin antibody, Rabbit polyclonal (Abcam Cat# ab11062). IP tested on rat brain by supplier. https://www.abcam.com/cofilin-1-antibody-ab11062.html. Previously used by Bamburg's lab to detect cofilin rods
Anti-actin antibody, Mouse monoclonal, clone C4 (Millipore Cat# MAB1501). This highly published monoclonal antibody is validated by supplier for use in ELISA, IC, IF,IH,IH(P) & WB, by using HeLa whole cell lysate as control. https://www.emdmillipore.com/US/en/product/Anti-Actin-Antibody-clone-C4,MM_NF-MAB1501
Anti-Hypoxyprobe antibody, Rabbit antisera (Hypoxyprobe Cat# HP3-1000Kit). Supplier found that there is very low background in mouse tissues when the Hypoxyprobe™ Plus Kit is combined with an anti-FITC secondary reagent. Pimonidazole is reductively activated in hypoxic cells and forms stable adducts with thiol (sulphydryl) groups in proteins, peptides and amino acids. FITC-MAb binds to these adducts allowing their detection by immunochemical means. http://site.hypoxyprobe.com/knowledge-center-articles/HP-Plus-Kit-Insert.pdf
Anti-mouse IgG antibody Alexa Fluor 647, Goat (Invitrogen Cat# A21237) https://www.thermofisher.com/order/genome-database/generatePdf?productName=Mouse%20IgG%20(H+L)%20Cross-Adsorbed&assayType=PRANT&detailed=true&productId=A-21237

Anti-mouse IgG antibody Alexa Fluor 488, Goat (Invitrogen Cat# A11001) https://www.thermofisher.com/antibody/product/Goat-anti-Mouse-IgG-H-L-Cross-Adsorbed-Secondary-Antibody-Polyclonal/A-11001
Anti-mouse IgG antibody Alexa Fluor 568, Goat (Invitrogen Cat# A11031) https://www.thermofisher.com/antibody/product/Goat-anti-Mouse-IgG-H-L-Highly-Cross-Adsorbed-Secondary-Antibody-Polyclonal/A-11031
Anti-rabbit IgG antibody Alexa Fluor 568, Goat (Invitrogen Cat# A11011) https://www.thermofisher.com/antibody/product/Goat-anti-Rabbit-IgG-H-L-Cross-Adsorbed-Secondary-Antibody-Polyclonal/A-11011
Anti-rabbit IgG antibody Alexa Fluor 488, Goat (Invitrogen Cat# A11008) https://www.thermofisher.com/antibody/product/Goat-anti-Rabbit-IgG-H-L-Cross-Adsorbed-Secondary-Antibody-Polyclonal/A-11008
Anti-rabbit IgG antibody Alexa Fluor 647, Goat (Invitrogen Cat# A21244) https://www.thermofisher.com/antibody/product/Goat-anti-Rabbit-IgG-H-L-Cross-Adsorbed-Secondary-Antibody-Polyclonal/A-21244

Animals and other research organisms

Policy information about studies involving animals; ARRIVE guidelines recommended for reporting animal research, and Sex and Gender in Research

Laboratory animals	Rat: Sprague Dawley Charles River cat# 400 [both M and F embryos from E19 time-pregnant female]; Mouse: C57BL/6J The Jackson Laboratory cat# 000664 [4 months old both M and F] Mouse: Thyl-YFP-H The Jackson Laboratory cat# 003782 [4 months old both M and F]. All mice were housed on a 12 dark/12 light cycle at temperatures of 20-26 °C (set point 22.8°C) with 30-70 % humidity (set point 50%).
Wild animals	No wild animals were used in the study
Reporting on sex	Findings do not apply to only one sex; sex was not considered in study design and no sex-based analysis was performed.
Field-collected samples	No field collected samples were used in the study.
Ethics oversight	All the following procedures involving animals were conducted in accordance with the US National Institutes of Health Guide for the Care and Use of Laboratory Animals and were approved by the Institutional Animal Care and Use Committee (IACUC) at UC San Diego (protocol # S07290) and Medical University of South Carolina (protocol # 3219).

Note that full information on the approval of the study protocol must also be provided in the manuscript.
